# Dynamic CT-based body composition analysis predicts surgical risk in Crohn’s disease with small bowel stenosis: a retrospective cohort study

**DOI:** 10.3389/fmed.2025.1642851

**Published:** 2025-09-18

**Authors:** Mengting Huang, Huan Wang, Shuo Huang, Qinyue Luo, Jinbo Gao, Ping Han, Liangru Zhu, Heshui Shi

**Affiliations:** ^1^Department of Radiology, Union Hospital, Tongji Medical College, Huazhong University of Science and Technology, Wuhan, China; ^2^Hubei Provincial Clinical Research Center for Precision Radiology and Interventional Medicine, Wuhan, China; ^3^Hubei Key Laboratory of Molecular Imaging, Wuhan, China; ^4^Division of Gastroenterology, Union Hospital, Tongji Medical College, Huazhong University of Science and Technology, Wuhan, China; ^5^Department of Gastrointestinal Surgery, Union Hospital, Tongji Medical College, Huazhong University of Science and Technology, Wuhan, China

**Keywords:** Crohn’s disease, body composition, skeletal muscle, CT, surgery, visceral adipose tissue

## Abstract

**Background:**

Reliable predictors of surgical risk in Crohn’s disease (CD) with small bowel stenosis are lacking. Longitudinal CT enterography (CTE) derived body composition parameters may improve risk stratification.

**Aims:**

To evaluate whether longitudinal CTE-derived body composition changes predict surgical risk in CD patients with small bowel stenosis.

**Methods:**

This retrospective cohort study analyzed 385 CD patients between January 2018 and June 2022 with paired CTE scans. High-risk patients (*n* = 96) required surgery for complications; low-risk patients (*n* = 289) achieved medical remission. Skeletal muscle (SM), subcutaneous adipose tissue (SAT), visceral adipose tissue (VAT), and intermuscular adipose tissue (IMAT) metrics at L3–L5 levels were measured and normalized by vertebral height. Gender-stratified analyzes and Cox regression identified predictors.

**Results:**

There were 289 cases in the low-risk group and 96 cases in the high-risk group. Interaction terms (time and gender) were tested, males showed significant reductions in L3–L4 skeletal muscle index (SMI) (*p* < 0.001), L3-L4 IMAT index (*p* < 0.001, *p* = 0.04), and L4-L5 VAT density (*p* = 0.008, *p* = 0.005). Independent predictors of surgical risk included SAT density at L5 level in baseline (*p* = 0.005), SMI at L3 level in follow up (*p* < 0.001), VAT/total adipose tissue index (VTR) (*p* = 0.004), delta SMI at L4 level (*p* < 0.001), age (*p* < 0.001), platelet count (*p* = 0.010), erythrocyte sedimentation rate (*p* < 0.001), and stenosis length (*p* = 0.001).

**Conclusion:**

Dynamic body composition parameters, particularly delta SMI and adipose tissue parameters, serve as valuable imaging biomarkers for predicting surgical necessity in CD patients with small bowel stenosis.

## 1 Introduction

Crohn’s disease (CD), a chronic inflammatory bowel disease characterized by relapsing-remitting inflammation (1), can result in progressive transmural inflammation and fibrosis. Approximately 30–50% of patients develop stenotic complications over time, substantially increasing surgical intervention risk (2, 3). Epidemiological studies indicate that approximately 25.6–40% of CD patients develop stricturing phenotypes within 10 years of diagnosis (4), with rates of 29–35% observed in Chinese patient cohorts (5, 6). Although effective treatments to induce mucosal healing may exist, approximately more than half of the patients still require surgical intervention within 10 years of diagnosis (7, 8). Bernstein et al. report that small bowel stenosis of CD is a critical determinant of disease progression and surgical outcomes (9). Current surgical decision-making relies predominantly on luminal narrowing severity and proximal dilation (10), parameters with limited predictive value for individual trajectories. While current imaging modalities excel in anatomical characterization of strictures, their ability to predict surgical urgency remains limited a gap that may contribute to suboptimal timing in part of surgical decisions (6).

Emerging evidence suggests that body composition has emerged as a potential indicator of disease progression and clinical outcomes in various chronic conditions (11–13). In CD, changes in body composition may reflect underlying inflammatory activity, nutritional status, and overall disease burden. The role of visceral adipose tissue (VAT) in colonic inflammation has been reported (14, 15), and its abnormal accumulation is often associated with a more severe disease phenotype and adverse prognosis in patients with CD (16, 17). Though baseline CT body composition analysis shows promise in cross-sectional studies (18), the prognostic value of dynamic body composition patterns remains unexplored in longitudinal CD management. Particularly, longitudinally quantified body composition metrics including temporal changes in visceral adipose tissue radiodensity and skeletal muscle mass may dynamically capture early metabolic perturbations predictive of subsequent clinical manifestations in CD.

Consequently, this study aimed to quantify dynamic changes in CT-derived body composition parameters in CD patients presenting with small bowel stenosis; to evaluate the independent and combined predictive value of serial body composition measures, alongside baseline clinical or biochemical markers and stenosis morphology, for predicting surgical risk; to develop and validate a clinically applicable risk-stratification tool incorporating these predictors; and to investigate the level-specific prognostic significance of body composition metrics across specific vertebral levels.

## 2 Methods

### 2.1 Study subjects

A total of 503 adult patients consecutively admitted to between January 2018 and June 2022 were included, with the following inclusion criteria: (i) received prior medical therapy for CD and subsequently confirmed small bowel stenosis by the gastroenterology department of our hospital (19); (ii) underwent at least two CT enterography (CTE) scans (denoted as Time1 and Time2 respectively). Included patients had established CD and developed new small bowel stenosis during follow-up. All were on ongoing medical therapy at the Time1. Time1 is the time of the CTE examination when intestinal stenosis was first detected, and the time interval between Time2 and Time1 should be between 6 and 12 months.

Exclusion criteria were as follows: (i) history of abdominal surgery (excluding appendectomy) prior to Time1 examination; (ii) incomplete clinical or imaging data; (iii) the interval between time1 and time2 is less than 6 months (i.e., imminent surgery candidates); (iv) diagnosis of intestinal stricture excluded by a senior radiologist upon review, or the stenotic intestinal segment not being located in the small intestine segment; (v) associated with diseases that can affect the distribution or function of adipose tissue such as malignancies or metabolic diseases (hyperthyroidism, diabetes).

**FIGURE 1:**
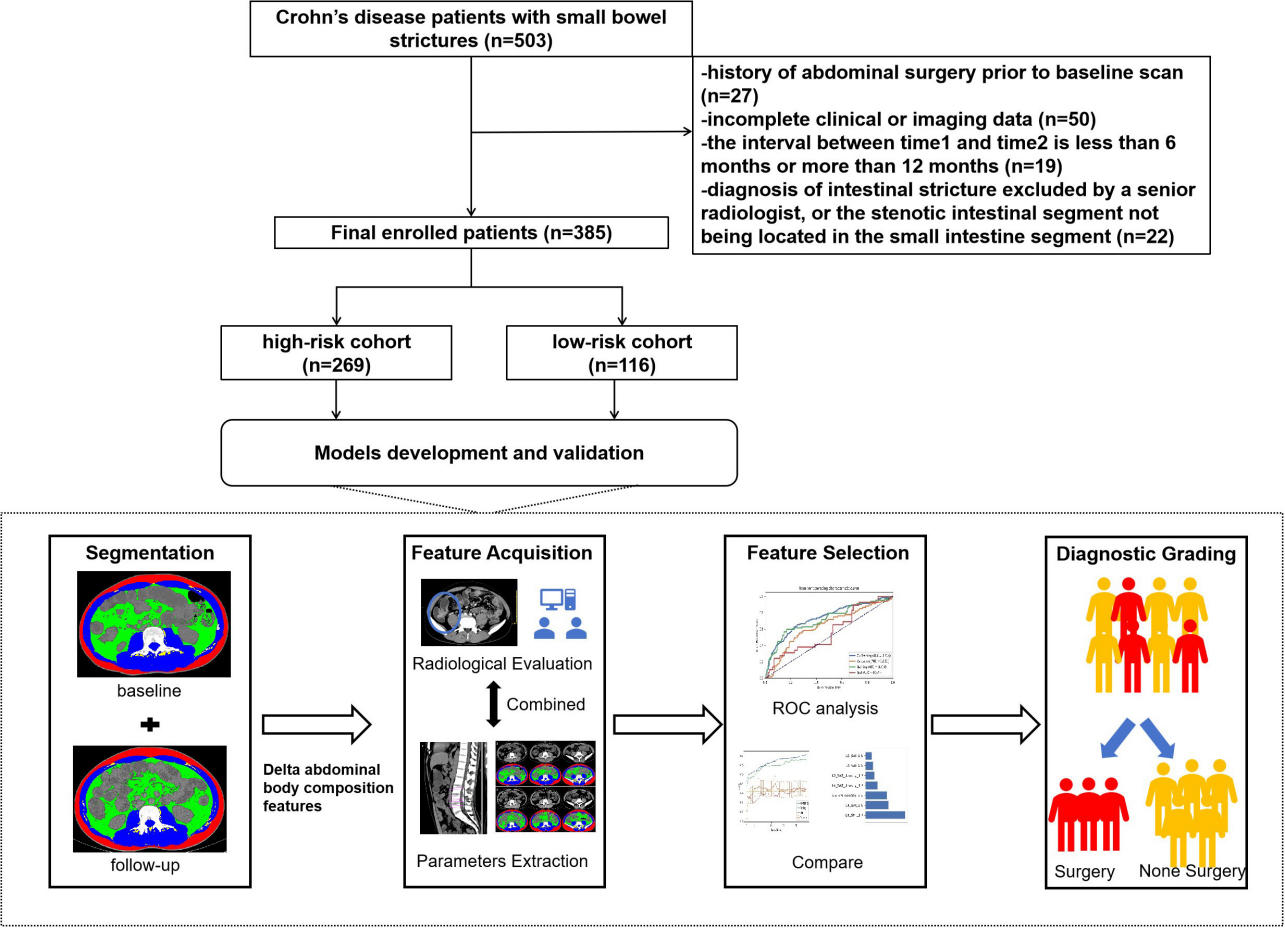
Patient selection flowchart. ROC: receiver operating characteristic.

Finally, 385 patients were included in this retrospective study, and the patient selection process is shown in the Figure 1. Clinical characteristics and treatment regimens, including age, sex, disease duration, Montreal classification, and laboratory parameters were recorded. The classification of small bowel CD is defined per the Consensus Statement on Endoscopic Diagnosis and Treatment for Small Bowel Crohn’s Disease, wherein: L1 denotes disease confined to the terminal ileum; L3 indicates involvement of both terminal ileum and colon; L4 denotes involvement of the upper gastrointestinal tract (Supplementary Material 1). Patients were classified as immunomodulators, biological, and combination therapy based on therapy prior to Time1.

To ensure robust validation of predictive models while preventing overfitting, an *a priori* cohort partitioning strategy was employed. Patients were randomly allocated to training and test cohorts at a 7:3 ratio for internal validation. The training set optimized predictive parameters weights through 10-fold cross-validation, while the test set assessed model discrimination and calibration in an independent population. This retrospective study was approved by the ethics committee of our hospital (No.0940-01), with a waiver of written informed consent.

### 2.2 Image acquisition

To achieve optimal bowel distension, all patients ingested 1,000–2,000 ml of 2.5% isosmotic mannitol solution within 1 h prior to CTE scanning. To reduce intestinal motility, 10-mg of racemic anisodamine hydrochloride was administered intramuscularly 15 min before the scan. CTE images were acquired using one of three CTE scanners (SOMATOM Definition AS+, SOMATOM Force, Philips Healthcare IQon spectral CT). The tube voltage was set at 120 kVp with automatic tube current modulation. Iodinated contrast agent was injected intravenously at a rate of 3.5 mL/s, followed by a saline flush at the same injection rate. Images were reconstructed at a slice thickness of 1.5 mm with an increment of 1.5 mm. The dose-length product (DLP) and volume CT dose index (CTDIvol) were also recorded, and the effective radiation dose was calculated by multiplying the DLP by the conversion factor (0.015 mSv/mGy.cm).

### 2.3 Image evaluation

Two radiologists, blinded to patients’ clinical history, independently reviewed baseline CTE images. Each small bowel segment meeting stricture criteria on baseline examination was analyzed, thus ensuring at least one confirmed stricture per patient. The narrow segment is defined as follows: the lumen diameter of the narrow segment is less than 50% of the diameter of the normal intestinal segment; the intestinal wall is thickened by at least 25% compared with the normal intestinal wall; and the proximal small intestine of the narrow segment is dilated by more than 3 cm (20). Radiological assessments included: (i) single vs. multiple luminal narrowing regions within strictures; (ii) presence of marked small bowel dilation greater than 4 cm (21, 22); (iii) imaging signs of active inflammation at stricture sites (e.g., bowel wall thickening, mucosal ulceration); (iv) degree of bowel wall enhancement (none, mild, moderate, severe); (v) comb sign; and (vi) periaortic findings such as mesenteric edema.

**FIGURE 2:**
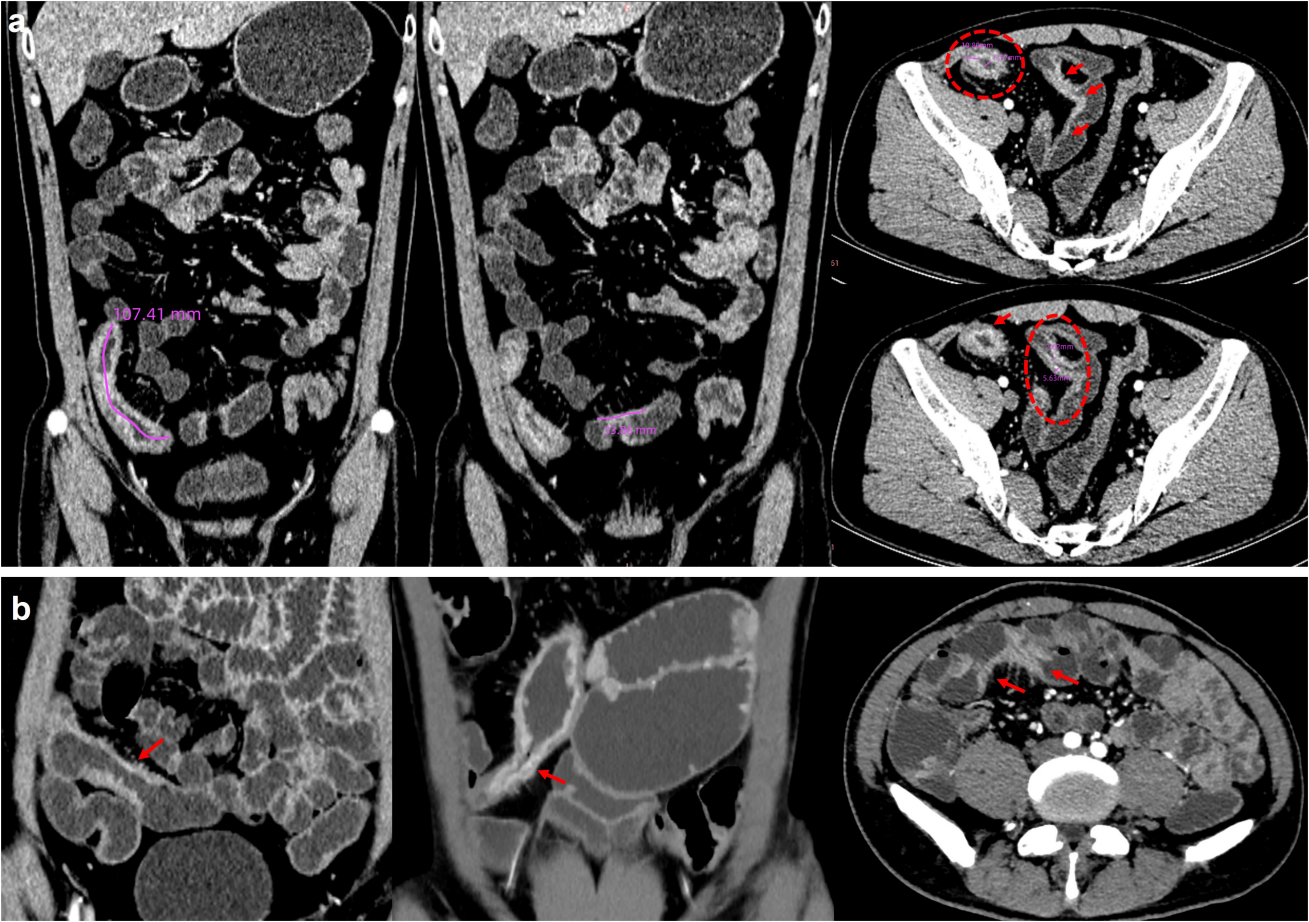
An example of imaging measurements and stricture morphology. **(a)** An example of imaging measurements: stricture length, narrowest luminal diameter, the maximum bowel wall thickness; **(b)** stricture morphology: strictures without inflammatory, strictures with inflammatory, and multifocal strictures.

The following parameters were measured (20, 22): (i) length of the abnormal small bowel segment; (ii) luminal diameter (at the narrowest point within the stricture); (iii) maximum bowel wall thickness (at the site of maximal luminal narrowing); (iv) maximum diameter of proximal small bowel dilation (in any plane) (Figure 2a). For patients with multiple adjacent stenosis small bowel segments, the entire involved small bowel segment encompassing adjacent strictures was included for length measurement. Each patient was categorized into four distinct morphological groups: strictures with inflammatory imaging findings, strictures without inflammatory imaging findings, single-focal strictures, and multifocal strictures (defined as adjacent strictures less than 3 cm apart) (20) (Figure 2b). Narrow parameters exhibiting overlapping characteristics were divided into two distinct categories, after which a dominant category was determined. This classification was established through consensus between two independent radiologists due to the absence of a reference standard. Subsequent analyzes focused on quantitatively derived imaging biomarkers due to subgroup heterogeneity.

### 2.4 Abdominal body composition parameter quantification

The distance from the superior edge of the first lumbar vertebra (L1) to the inferior edge of the fifth lumbar vertebra (L5) was measured in the coronal plane and defined as the L1–L5 height. Three slices were evenly selected at the level of the third to fifth lumbar vertebrae for each patient, and a total of 9 slices were used for subsequent analysis (13) (Supplementary Figure 2). Body composition distribution varies craniocaudally due to differential fat deposition patterns and muscle morphology. The L3 level is widely recognized as a validated surrogate for whole-abdomen body composition, while L4–L5 levels capture pelvic and trunk muscle-fat dynamics specific to inflammatory conditions like CD. Skeletal muscle (SM), subcutaneous adipose tissue (SAT), VAT, and intermuscular adipose tissue (IMAT) were segmented using a semi-automatic method in SlicerOmatic. The main steps were to outline the SM by setting a threshold of −29 to +150 HU, to outline the SAT and IMAT by setting a threshold of −190 to −30 HU, and to outline the VAT by setting a threshold of −150 to −50 HU (13, 23, 24) (Supplementary Figure 3). The area and mean attenuation of SM, SAT, VAT, and IMAT were quantified, respectively. The correction was carried out by another radiologist, mainly including the inspection and correction of the preliminary results. The standardized indices SM index (SMI), subcutaneous adipose index (SAI), visceral adipose index (VAI), and intermuscular adipose index (IMAI) were obtained by dividing the area of each component (SM, SAT, VAT, IMAT) by the square of the vertebral height (25, 26). Additionally, the VAT/SAT ratio (VSR), and VAT/total adipose tissue index (VTR) (13) were calculated to reflect the proportional distribution of VAT, as detailed in Supplementary Material 2.

To evaluate inter-observer and intra-observer reproducibility of the parameters, 40 patients randomly selected from the training cohort underwent two validation sessions at a 1-month interval by a second radiologist using identical tools and environmental settings. Inter-rater reliability was determined via intraclass correlation coefficients (ICCs) calculated with a two-way random-effects model.

### 2.5 Delta abdominal body composition parameters

For patients with two CTE scans, abdominal body composition parameters were extracted from both time points. Delta abdominal body composition parameters were defined as the relative net change between the two time points, calculated as: Delta abdominal body composition = (Parameters_Time2_ - Parameters_Time1_)/Parameters_Time1_. Negative values indicate a decrease in the parameter over time, while positive values indicate an increase.

### 2.6 Follow-up and definition

The patients were followed up until September 30, 2024. The primary endpoint events comprised surgical interventions for small bowel strictures occurring before the study termination date. Stricture-specific interventions: small-bowel resections or endoscopic balloon dilatations indicated for the treatment of symptomatic strictures. Subjects undergoing any such intervention were categorized into the high-risk group; others comprised the low-risk group. Overall survival (OS) time was defined as the interval from the initial CT scan (Time1) to the occurrence of endpoint events or the cutoff date, served as the primary time-to-event endpoint for prognostic evaluation.

### 2.7 Statistical analysis

All statistical analyzes were performed using R (version 4.4.3). Continuous variables were expressed as mean ± standard deviation (SD), while categorical variables were presented in the form of counts and percentages. Paired or unpaired *t*-tests were employed to evaluate intergroup differences. The Spearman coefficient was used to quantify the correlations between body composition indices, and the Wilcoxon rank-sum test was used to evaluate the distribution differences of body composition indices. The Kappa coefficient was used to assess the agreement between the two radiologists in each CT evaluation, and a Kappa coefficient greater than 0.75 indicated good agreement.

The Cox proportional hazards regression was designed to predict surgery-requiring disease progression in CD patients with small bowel stenosis, using baseline and dynamic body composition parameters. Variables with significance (two-tailed *P* < 0.05) in univariate analysis and variance inflation factors <5 were included in the final multivariable model. Delta values were calculated as (Time 2 - Time 1)/Time 1), with negative values indicating decrease and positive values increase. In Cox models, HR < 1.0 for delta variables denotes protection associated with metric increase. A nomogram was constructed using significant predictors identified by multivariate analysis. Discrimination was assessed using time-dependent ROC curves, with AUC reported for 12 months, 18 months and more than 21 months outcomes.

Given established sex-based differences in body composition parameters, participants were stratified into two distinct subgroups: males and females. All subsequent survival analyzes, including univariate and multivariate Cox proportional hazards regression, were performed independently for each subgroup using the original, non-pooled values of body composition metrics. This stratification ensured that gender-specific predictors of surgical risk were identified while mitigating confounding effects.

## 3 Results

### 3.1 Clinical characteristics and CTE parameters

**FIGURE 3:**
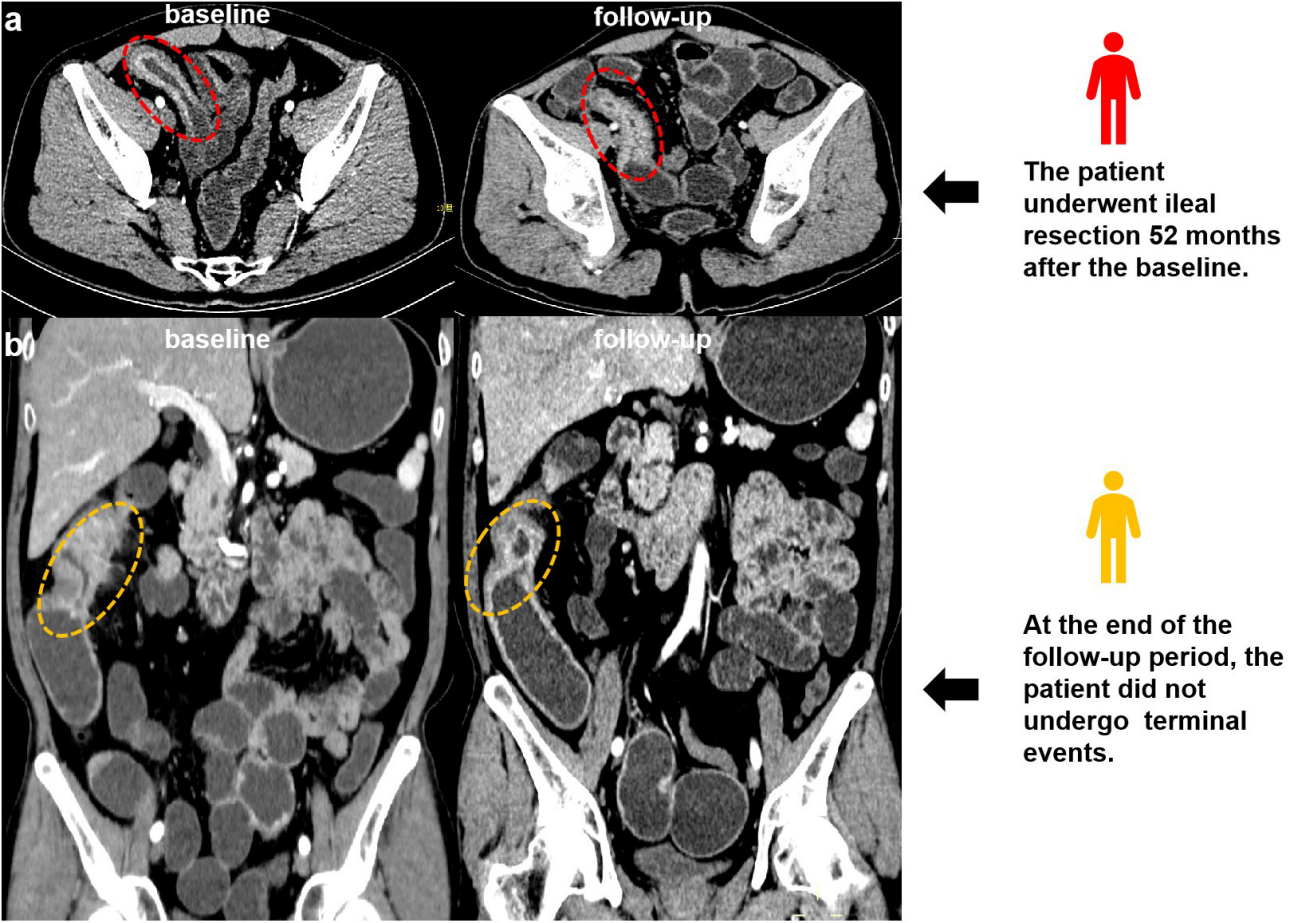
**(a)** Examples of imaging: A 35-year-old man with small bowel stricture and the wall thickening is observed in the baseline and follow-up (red circle). The patient underwent ileal resection 52 months after the baseline computed tomography enterography (CTE). **(b)** Examples of imaging: A 49-year-old man with small bowel stricture and the wall thickening is observed in the baseline and follow-up (yellow circle). At the end of the follow-up period, the patient did not undergo endpoint events.

Baseline clinical and demographic characteristics of the cohort are presented in Table 1. Of the 385 enrolled patients (male, 304/385, 78.9%; mean age, 33.68 ± 10.44 years). By the study cutoff date (September 30, 2024), 96 patients (24.9%) met endpoint events criteria (detailed in Section 2.6) and were classified into the high-risk group (examples of imaging, Figure 3a), while 289 patients (75.1%) remained event-free and were assigned to the low-risk group (examples of imaging, Figure 3b).

**TABLE 1:** Baseline characteristics of Crohn’s disease patients.

Characteristics	All patients N = 385	High risk N = 96	Low risk N = 289	*P-*value
Age (year)	33.7 ± 10.4	37.6 ± 10.4	32.3 ± 10.1	0.001
Male (%)	304 (78.9%)	76 (79.2%)	228 (78.9%)	0.955
Female (%)	81 (21.1%)	20 (20.8%)	61 (21.1%)	
BMI (kg/m^2^)	18.9 ± 3.1	18.7 ± 2.9	18.9 ± 3.1	0.578
Disease duration (year)	2.67 ± 0.74	2.74 ± 0.82	2.65 ± 0.71	0.315
Leukocyte (10^9^/L)	7.3 ± 2.2	7.3 ± 2.8	7.3 ± 2.0	0.857
Hemoglobin (g/L)	119.4 ± 21.8	117.7 ± 19.9	120.0 ± 22.4	0.382
Platelet (10^9^/L)	218.5 ± 51.3	238.7 ± 44.2	211.8 ± 51.8	0.001
Hematocrit (%)	38.9 ± 5.4	38.7 ± 5.3	39.0 ± 5.5	0.709
CRP (mg/L)	40.5 ± 39.3	48.5 ± 57.9	37.8 ± 30.4	0.021
ESR (mm/h)	8.7 ± 5.0	11.6 ± 4.8	7.7 ± 4.7	0.001
FC (μg/g)	261.2 ± 173.1	371.4 ± 201.3	224.6 ± 145.5	0.001
Albumin (g/L)	37.7 ± 8.1	34.7 ± 9.2	38.7 ± 7.5	0.001
L1 (N%)	49 (12.7%)	12 (12.5%)	37 (12.8%)	0.547
L3 (N%)	336 (87.3%)	84 (87.5%)	252 (87.2%)	
^#^L4 (N%)	26 (6.7%)	9 (9.3%)	17 (5.9%)	
P (N%)	201(52.2%)	54 (56.3%)	147 (50.9%)	0.360
Immunomodulators (N%)	101 (26.2%)	22 (22.9%)	79 (27.3%)	0.134
Biologics (N%)	225 (58.4%)	64 (66.7%)	161 (55.7%)	
Combination (N%)	59 (15.3%)	10 (10.4%)	49 (17.0%)	
CTE parameters	-	-	-	-
Length (mm)	35.7 ± 16.9	46.8 ± 16.2	32.0 ± 15.5	0.001
Diameter (mm)	7.9 ± 3.4	7.7 ± 3.7	8.0 ± 3.3	0.589
Proximal (mm)	31.7 ± 15.4	32.6 ± 12.2	31.4 ± 16.4	0.512
Thickness (mm)	7.4 ± 2.3	7.4 ± 2.0	7.4 ± 2.4	0.972
Comb sign (N%)	236 (61.2%)	75 (78.1%)	161 (55.7%)	0.001
Lymph nodes (N%)	320 (83.1%)	85 (88.0%)	235 (81.3%)	0.101
Stricture with inflammation (N%)	274 (71.2%)	74 (77.1%)	200 (69.2%)	0.140
Stricture without inflammation (N%)	111 (28.8%)	22 (22.9%)	89 (30.8%)	
Single strictures (N%)	277 (71.9%)	67 (69.8%)	210 (72.7%)	0.587
Multifocal strictures (N%)	108 (28.1%)	29 (30.2%)	79 (27.3%)	

BMI, body mass index; CRP, C-reactive protein; ESR, Erythrocyte sedimentation rate; FC, Fecal calprotectin; L1, ileal; L3, ileocolonic; L4, upper digestive tract; ^#^was coexist with other disease localization phenotypes. P, perianal disease; CTE, CT enterography.

The high-risk group exhibited a significantly older mean age compared to the low-risk group (*p* = 0.001). No significant differences were observed in sex distribution (*p* = 0.955), disease duration (*p* = 0.315), or disease localization phenotypes (*p* = 0.360). A total of 201 (52.2%) patients presented with perianal lesions too, in addition to small bowel strictures. Significant elevations in inflammatory biomarkers were observed in the high-risk group. This included higher fecal calprotectin (FC) levels (371.4 ± 201.3 μg/g vs. 224.6 ± 145.5 μg/g; *p* = 0.001), C-reactive protein (CRP) (48.5 ± 57.9 mg/L vs. 37.8 ± 30.4 mg/L; *p* = 0.021), and erythrocyte sedimentation rate (ESR) (11.6 ± 4.8 mm/h vs. 7.7 ± 4.7 mm/h; *p* = 0.001). Conversely, serum albumin levels were significantly lower in the high-risk group (*p* = 0.001). Platelet counts were also significantly higher in the high-risk cohort (*p* = 0.001).

Cross-sectional CTE imaging revealed that high-risk patients demonstrated significantly longer affected bowel segments on CTE compared to low-risk patients (*p* = 0.001). The prevalence of the comb sign, indicating prominent vasa recta engorgement, was markedly higher in the high-risk group (78.1% vs. 55.7%, *p* = 0.001). Other CTE parameters, including stricture diameter, proximal dilation, bowel wall thickness, presence of lymphadenopathy, stricture type (inflammatory vs. fibrotic), and stricture focality (single vs. multifocal), showed no significant differences between the two groups (*p* > 0.05). Gender-stratified analysis confirmed no significant differences in stricture distribution or morphology (Supplementary Table 1). The mean CTDIvol and DLP for CTE were 14.2 mGy (range 8.2–22.4 mGy) and 830.7 mGy.cm (range 413.9–1438.6 mGy.cm), and the average effective dose was 12.5 mSv (range 6.2–21.6 mSv).

To validate the robustness of our predictive models, we rigorously compared baseline and longitudinal characteristics between the training (*n* = 269) and test (*n* = 116) cohorts. At baseline (Time1), no statistically significant differences were observed in baseline clinical characteristics, including age, sex, disease duration, Montreal classification, inflammatory biomarkers, or stricture morphology (*p* > 0.05; Supplementary Table 2).

### 3.2 Temporal changes in body composition parameters of all patients

**FIGURE 4:**
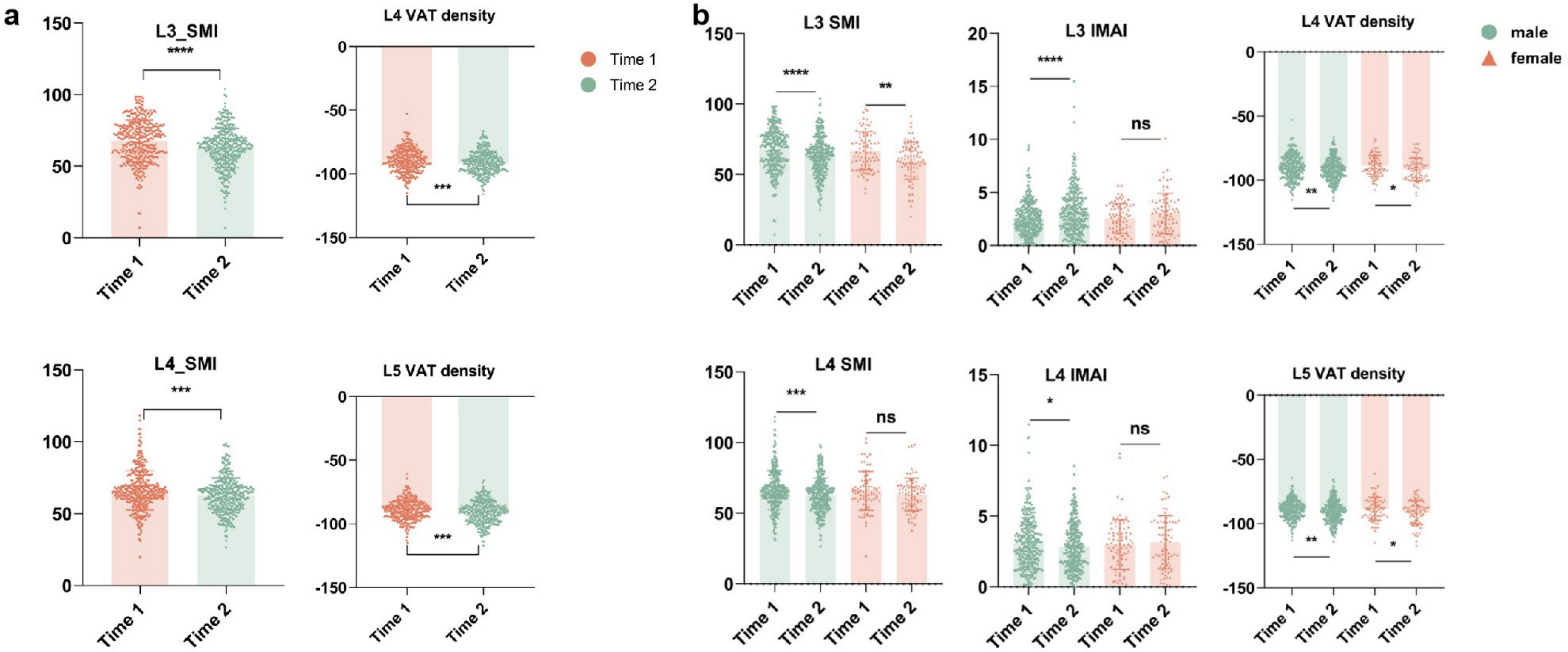
**(a)** Time-course changes in body composition in patients with Crohn’s disease. **(b)** Time-course changes in body composition in male and female subgroups of Crohn’s disease patients. SMI, skeletal muscle index; VAT, visceral adipose tissue. *P < 0.05, **P < 0.01, ***P < 0.001, ****P < 0.0001.

In this study, SMI (L3–L4 level, *p* < 0.001) and VAT density (L4–L5 level, *p* < 0.001) showed significant reductions at 6–12 months follow up (Time 2) compared with baseline (Time 1) (Figure 4a). Interaction terms (time and gender) were tested, in males, SMI (L3–L4 level, *p* < 0.001), IMAI (L3–L4 level, *p* < 0.001, *p* = 0.04), and VAT density (L4–L5, *p* = 0.008, *p* = 0.005) demonstrated significant change s. In females, SMI (L3 level, *p* = 0.001) and VAT density (L4–L5 level, *p* = 0.018, *p* = 0.026) showed significant reductions (Figure 4b and Table 2). Longitudinal body composition parameters measured at follow-up (Time2) also demonstrated homogeneity between cohorts across all vertebral levels (L3–L5), with no significant differences in SMI, VAT density, or IMAI (all *p* > 0.05; Supplementary Table 3).

**TABLE 2:** Longitudinal alterations of body composition parameters in Crohn’s disease patients.

Parameters		Male			Female		
		Time 1	Time 2	*P*-value	Time 1	Time 2	*P*-value
L3	IMAI (10^2^ cm^2^/m^2^)	2.62 ± 1.55	3.23 ± 2.17	<0.001	2.63 ± 1.47	3.03 ± 1.90	0.074
	IMAT density (HU)	−59.44 ± 6.17	−60.58 ± 5.75	0.012	−59.77 ± 5.88	−60.01 ± 5.71	0.767
	SAI (10^2^ cm^2^/m^2^)	43.87 ± 23.02	42.57 ± 16.39	0.388	43.04 ± 20.79	40.78 ± 17.09	0.405
	SAT density (HU)	−91.65 ± 12.90	−91.05 ± 13.83	0.537	−89.50 ± 14.15	−92.22 ± 13.80	0.188
	SMI (10^2^ cm^2^/m^2^)	68.05 ± 14.70	63.34 ± 13.72	<0.001	67.06 ± 13.24	60.36 ± 12.85	0.001
	SM density (HU)	42.19 ± 7.64	41.53 ± 7.60	0.226	43.23 ± 8.15	42.26 ± 6.98	0.333
	VAI (10^2^ cm^2^/m^2^)	37.83 ± 22.17	41.34 ± 17.94	0.014	40.34 ± 23.56	38.20 ± 18.47	0.403
	VAT density (HU)	−89.70 ± 9.53	−90.63 ± 9.06	0.179	088.93 ± 9.95	−90.14 ± 8.58	0.364
	VSR	0.83 ± 0.42	0.98 ± 0.48	<0.001	0.84 ± 0.47	0.93 ± 0.51	0.140
	VTR	0.42 ± 0.12	0.43 ± 0.12	0.201	0.41 ± 0.12	0.41 ± 0.12	0.927
L4	IMAI (10^2^ cm^2^/m^2^)	3.08 ± 1.82	2.83 ± 1.64	0.403	3.03 ± 1.80	3.16 ± 1.86	0.593
	IMAT density (HU)	−60.11 ± 6.21	−60.60 ± 5.68	0.322	−59.85 ± 6.47	−60.73 ± 5.90	0.322
	SAI (10^2^ cm^2^/m^2^)	43.29 ± 18.55	43.25 ± 13.06	0.978	43.67 ± 17.94	43.45 ± 16.21	0.931
	SAT density (HU)	−92.89 ± 13.62	−92.84 ± 12.71	0.960	−92.61 ± 12.48	−91.78 ± 12.44	0.623
	SMI (10^2^ cm^2^/m^2^)	66.39 ± 13.72	62.79 ± 12.03	<0.001	65.67 ± 13.31	63.73 ± 11.61	0.280
	SM density (HU)	42.90 ± 7.51	42.97 ± 7.34	0.894	42.40 ± 6.91	42.85 ± 7.08	0.660
	VAI (10^2^ cm^2^/m^2^)	39.56 ± 18.94	41.56 ± 13.17	0.102	37.35 ± 22.34	39.02 ± 13.69	0.535
	VAT density (HU)	−89.99 ± 9.34	−91.68 ± 8.46	0.009	−88.75 ± 8.21	−91.44 ± 8.69	0.015
	VSR	0.73 ± 0.36	0.72 ± 0.35	0.686	0.73 ± 0.38	0.77 ± 0.33	0.273
	VTR	0.39 ± 0.11	0.39 ± 0.11	0.986	0.38 ± 0.11	0.40 ± 0.11	0.112
L5	IMAI (10^2^ cm^2^/m^2^)	4.92 ± 2.55	4.62 ± 2.18	0.120	4.88 ± 2.59	4.78 ± 2.15	0.760
	IMAT density (HU)	−65.85 ± 7.65	−65.57 ± 7.94	0.627	−65.28 ± 6.86	−65.67 ± 7.36	0.684
	SAI (10^2^ cm^2^/m^2^)	42.51 ± 16.28	39.11 ± 12.64	0.002	42.85 ± 15.31	41.54 ± 13.44	0.545
	SAT density (HU)	−90.73 ± 13.09	−91.68 ± 13.49	0.315	−88.97 ± 14.80	−91.35 ± 13.91	0.234
	SMI (10^2^ cm^2^/m^2^)	62.58 ± 11.56	61.66 ± 10.88	0.280	61.37 ± 12.83	62.37 ± 10.47	0.534
	SM density (HU)	42.85 ± 7.07	41.27 ± 7.44	0.001	42.26 ± 6.92	40.96 ± 6.59	0.158
	VAI (10^2^ cm^2^/m^2^)	35.68 ± 15.44	34.40 ± 14.98	0.238	33.23 ± 14.86	36.44 ± 13.52	0.061
	VAT density (HU)	−89.26 ± 7.34	−90.98 ± 8.51	0.005	−88.29 ± 8.79	−91.14 ± 9.06	0.019
	VSR	0.69 ± 0.31	0.67 ± 0.33	0.413	0.66 ± 0.28	0.66 ± 0.29	0.842
	VTR	0.36 ± 0.09	0.35 ± 0.10	0.314	0.35 ± 0.09	0.35 ± 0.09	0.908

IMAI, intermuscular adipose index; IMAT, intermuscular adipose tissue; SAI, subcutaneous adipose index; SAT, subcutaneous adipose tissue; SMI, skeletal muscle index; SM, skeletal muscle; VAI, visceral adipose index; VAT, visceral adipose tissue; VAR, VAT/SAT ratio; VTR, VAT/total adipose tissue index.

**FIGURE 5:**
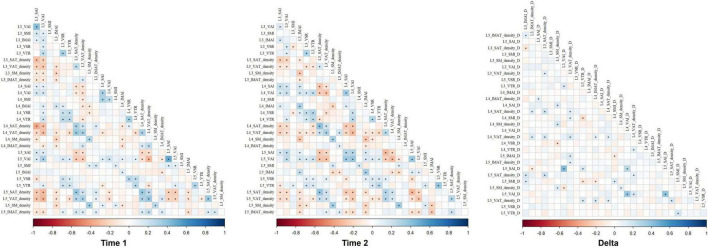
The correlations of abdominal body composition parameters values across all vertebral levels in Time 1, Time 2, and delta. IMAI, intermuscular adipose index; IMAT, intermuscular adipose tissue; SAI, subcutaneous adipose index; SAT, subcutaneous adipose tissue; SMI, skeletal muscle index; SM, skeletal muscle; VAI, visceral adipose index; VAT, visceral adipose tissue; VAR, VAT/SAT ratio; VTR, VAT/total adipose tissue index.

Additionally, we observed an interesting phenomenon where there was a high correlation between the same body composition characteristics measured at levels L3, L4, and L5 at both Time1 and Time2. Specifically, correlations between adjacent vertebral levels (L3/L4, L4/L5) were marginally higher than those between non-adjacent levels (L3–L5). Notably, correlations of delta abdominal body composition parameters values across all vertebral levels were slightly lower than those between Time 1 and Time 2 measurements for the same level (Figure 5). These correlations support the spatial reliability of lumbar-level assessments while underscoring that temporal changes in body composition are regionally heterogeneous.

### 3.3 Assessment of follow-up information

By the study cutoff date (September 30, 2024), 96 patients (24.9%) met endpoint events criteria (as detailed in Section 2.6) and were classified into the high-risk group, while 289 patients (75.1%) remained event-free and were assigned to the low-risk group. The mean follow-up duration was 44.67 ± 1.16 months (range: 6–60 months). Univariate and multivariate Cox proportional hazards analyzes were conducted to identify predictors of adverse outcomes within the cohort. Upon incorporating clinical and CT imaging assessment variables, nine independent predictors of disease progression were identified.

Baseline assessment identified several parameters exhibiting univariate associations with survival. Significant univariate predictors included: increased SAT density at L3–L5 level (*p* < 0.001, *p* < 0.001, *p* = 0.021), SMI at L4 level (*p* = 0.009), VAT density at L4 level (*p* = 0.018), decreased SM density at L3 and L5 level (*p* = 0.022, *p* = 0.008), SAI at L4–L5 level (*p* = 0.014, *p* = 0.012), and VAI at L5 level (*p* = 0.012). Following covariate adjustment, only SAT density at L5 level retained independent prognostic significance. Elevated SAT density at L5 level was positively associated with increased hazard (HR 1.026; 95% CI 1.008–1.044; *p* = 0.005). Time 2 measurements identified significant univariate predictors comprising: increased SMI at L3 (*p* < 0.001), SAT density at L3 and L5 level (*p* = 0.016, *p* = 0.024), VAT density at L3–L5 level (*p* = 0.006, *p* < 0.001, *p* < 0.001), IMAT density at L4–L5 level (*p* < 0.001, *p* = 0.043), decreased VAI at L3–L5 level (*p* = 0.005, *p* = 0.042, *p* = 0.036), and VTR at L4 and L5 level (*p* = 0.036, *p* = 0.017). Multivariate analysis established SMI at L3 level (HR 1.042, 95% CI 1.023–1.061, *p* < 0.001) and VTR at L4 level (HR 0.022, 95% CI 0.002–0.304, *p* = 0.004) as significant independent predictors. Elevated SMI at L3 level was associated with increased hazard, whereas reduced VTR at L4 level demonstrated a protective effect.

Significant univariate associations for longitudinal changes included decreased delta SMI at L4 level (*p* = 0.001), decreased delta VAT density at L5 level (*p* = 0.013), and decreased delta VTR at L5 level (*p* = 0.020). The magnitude of reduction in delta SMI at L4 level was identified as a highly significant independent predictor in multivariate analysis (HR 0.100, 95% CI 0.028–0.354, *p* < 0.001). Significant clinical predictors included advanced age (*p* < 0.001), elevated platelet count (*p* = 0.003), increased ESR (*p* < 0.001), higher fecal calprotectin (*p* < 0.001), and reduced serum albumin (*p* < 0.001). Multivariate analysis incorporating clinical variables confirmed the independent prognostic significance of advanced age (HR 1.053, 95% CI 1.026–1.079, *p* < 0.001), elevated platelet count (HR 1.007, 95% CI 1.002–1.013, *p* = 0.010), increased ESR (HR 1.110, 95% CI 1.053–1.171, *p* < 0.001), and reduced serum albumin (HR 0.957, 95% CI 0.923–0.993, *p* = 0.018). Increased length of the abnormal small bowel segment was significantly associated with worse survival (*p* < 0.001). This parameter remained a significant independent predictor of adverse outcome following multivariate adjustment (HR 1.030, 95% CI 1.012–1.049, *p* = 0.001). Due to space constraints, Table 3 presents the statistically significant indicators (*p* < 0.05) from the univariate analysis for predicting high risk in CD patients within the training cohort. The complete results of both univariate and multivariate analyzes are provided in Supplementary Material 5.

**TABLE 3:** Univariate and multivariate survival analysis for predicting high risk patients.

Parameters		Univariate analysis			Multivariate analysis		
		β	*P*-value	HR (95% CI)	β	*P*-value	HR (95% CI)
Time 1	L3 SAT density	0.030	<0.001	1.030 (1.013–1.048)			
	L3 SM density	−0.035	0.022	0.965 (0.936–0.995)			
	L4 SAI	−0.018	0.014	0.982 (0.969–0.996)			
	L4 SMI	0.021	0.009	1.021 (1.005–1.037)			
	L4 SAT density	0.030	<0.001	1.031 (1.014–1.048)			
	L4 VAT density	0.034	0.018	1.035 (1.006–1.064)			
	L5 SAI	−0.018	0.012	0.982 (0.968–0.996)			
	L5 VAI	−0.022	0.012	0.979 (0.962–0.995)			
	L5 SAT density	0.021	0.021	1.021 (1.003–1.039)	0.026	0.005	1.026 (1.008–1.044)
	L5 SM density	−0.049	0.008	0.952 (0.918–0.987)			
Time 2	L3 VAI	−0.020	0.005	0.980 (0.966–0.994)			
	L3 SMI	0.057	<0.001	1.059 (1.039–1.080)	0.041	<0.001	1.042 (1.023–1.061)
	L3 SAT density	0.020	0.016	1.020 (1.004–1.037)			
	L3 VAT density	0.036	0.006	1.036 (1.010–1.063)			
	L4 VAI	−0.019	0.042	0.982 (0.964–0.999)			
	L4 VTR	−2.550	0.036	0.078 (0.007–0.842)	−3.804	0.004	0.022 (0.002–0.304)
	L4 SAT density	0.021	0.023	1.022 (1.003–1.041)			
	L4 VAT density	0.054	<0.001	1.055 (1.027–1.084)			
	L4 IMAT density	0.075	<0.001	1.078 (1.034–1.124)			
	L5 VAI	−0.018	0.036	0.983 (0.967–0.999)			
	L5 VTR	−3.015	0.017	0.049 (0.004–0.582)			
	L5 SAT density	0.020	0.024	1.020 (1.003–1.038)			
	L5 VAT density	0.067	<0.001	1.069 (1.038–1.101)			
	L5 IMAT density	0.033	0.043	1.034 (1.001–1.067)			
Delta	L4 SMI	−1.691	0.001	0.184 (0.067–0.506)	−2.303	<0.001	0.100 (0.028–0.354)
	L5 VTR	−0.864	0.020	0.422 (0.204–0.873)			
	L5 VAT density	−2.645	0.013	0.071 (0.009–0.574)			
Clinical	Age	0.043	<0.001	1.044 (1.022–1.067)	0.051	<0.001	1.053 (1.026–1.079)
	Platelet	0.008	0.003	1.008 (1.003–1.013)	0.007	0.010	1.007 (1.002–1.013)
	ESR	0.124	<0.001	1.132 (1.080–1.186)	0.105	<0.001	1.110 (1.053–1.171)
	Calprotectin	0.003	<0.001	1.003 (1.002–1.004)			
	Albumin	−0.050	<0.001	0.951 (0.924–0.979)			
CTE	Length	0.051	<0.001	1.053 (1.035–1.070)	0.030	0.001	1.030 (1.012–1.049)

SAT, subcutaneous adipose tissue; SMI, skeletal muscle index; VAI, visceral adipose index; VAT, visceral adipose tissue; IMAT, intermuscular adipose tissue; SM, skeletal muscle; VTR, VAT/total adipose tissue index; ESR, erythrocyte sedimentation rate; HR: Hazards Ratio; CI, confidence interval; CTE, CT enterography. Complete lists of all variables analyzed (including non-significant covariates) are provided in Supplementary Material 5.

Considering the gender differences, we conducted subgroup analyzes for males and females separately. In male cohort, baseline assessment identified several parameters exhibiting univariate associations with survival. Significant univariate predictors included: increased SAT density at L3–L4 level (*p* = 0.022, *p* = 0.006), SMI at L4–L5 level (*p* = 0.005, *p* = 0.016), decreased SM density at L5 level (*p* = 0.009) and SAI at L5 level (*p* = 0.044). Time 2 measurements identified significant univariate predictors comprising: increased SMI at L3 level (*p* < 0.001), VAT density at L3–L5 level (all *p* < 0.001), SAT density at L4–L5 level (all *p* < 0.001), IMAT density at L4–L5 level (all *p* < 0.001), decreased VAI at L3–L5 level (*p* < 0.001, *p* = 0.044, *p* = 0.005), IMAI at L3–L4 level (*p* = 0.035, *p* = 0.040), and VTR at L5 level (*p* = 0.032). Following covariate adjustment, only SM density at L5 level in Time 1 (HR 0.944; 95% CI 0.914–0.976; *p* < 0.001), SMI at L3 level in Time 2 (HR 1.056; 95% CI 1.036–1.076; *p* < 0.001), age (HR 1.038; 95% CI 1.017–1.060; *p* < 0.001), ESR (HR 1.073; 95% CI 1.025–1.122; *p* = 0.002), and length of the abnormal small bowel segment (HR 1.037; 95% CI 1.022–1.053; *p* < 0.001) retained independent prognostic significance (Table 4).

**TABLE 4:** Multivariate survival analysis results of Crohn’s disease patients in male.

Parameters		Univariate analysis			Multivariate analysis		
		β	*P-*value	HR (95% CI)	β	*P*-value	HR (95% CI)
Time 1	L3 SAT density	0.020	0.022	1.020 (1.003–1.038)			
	L4 SMI	0.021	0.005	1.021 (1.006–1.036)			
	L4 SAT density	0.022	0.006	1.023 (1.007–1.039)			
	L5 SAI	−0.014	0.044	0.986 (0.973–0.999)			
	L5 SMI	0.026	0.016	1.027 (1.005–1.049)			
	L5 SM density	−0.042	0.009	0.959 (0.929–0.990)	−0.057	<0.001	0.944 (0.914–0.976)
Time 2	L3 VAI	−0.023	<0.001	0.977 (0.964–0.990)			
	L3 SMI	0.055	<0.001	1.057 (1.038–1.076)	0.054	<0.001	1.056 (1.036–1.076)
	L3 IMAI	−0.127	0.035	0.881 (0.783–0.991)			
	L3 VAT density	0.042	<0.001	1.043 (1.019–1.067)			
	L4 VAI	−0.018	0.044	0.982 (0.964–0.999)			
	L4 IMAI	−0.153	0.040	0.858 (0.741–0.993)	−0.333	<0.001	0.717 (0.600–0.857)
	L4 SAT density	0.027	0.002	1.027 (1.010–1.045)			
	L4 VAT density	0.065	<0.001	1.068 (1.041–1.095)			
	L4 IMAT density	0.085	<0.001	1.089 (1.049–1.131)			
	L5 VAI	−0.021	0.005	0.979 (0.964–0.994)			
	L5 VTR	−2.549	0.032	0.078 (0.008–0.807)			
	L5 SAT density	0.024	0.006	1.024 (1.007–1.042)			
	L5 VAT density	0.072	<0.001	1.074 (1.046–1.103)	0.054	<0.001	1.055 (1.029–1.082)
	L5 IMAT density	0.051	<0.001	1.052 (1.022–1.084)			
Delta	L3 VAT density	−2.384	0.007	0.092 (0.016–0.516)			
	L4 SMI	−1.278	0.004	0.278 (0.116–0.671)			
	L4 VAT density	−2.086	0.027	0.124 (0.019–0.793)			
	L4 IMAT density	−2.691	0.002	0.068 (0.012–0.368)			
	L5 SMI	−0.982	0.036	0.375 (0.150–0.938)			
	L5 VAT density	−3.863	<0.001	0.021 (0.003–0.143)			
	L5 IMAT density	−1.803	0.027	0.165 (0.033–0.819)			
Clinical	Age	0.035	<0.001	1.035 (1.015–1.056)	0.038	<0.001	1.038 (1.017–1.060)
	Platelet	0.008	<0.001	1.008 (1.003–1.013)			
	C-reactive protein	0.006	0.015	1.006 (1.001–1.011)			
	ESR	0.111	<0.001	1.117 (1.069–1.167)	0.070	0.002	1.073 (1.025–1.122)
	Calprotectin	0.003	<0.001	1.003 (1.002–1.004)			
	Albumin	−0.037	0.009	0.964 (0.938–0.991)			
CTE	Length	0.043	<0.001	1.044 (1.030–1.057)	0.036	<0.001	1.037 (1.022–1.053)

IMAI, intermuscular adipose index; SAT, subcutaneous adipose tissue; SMI, skeletal muscle index; VAI, visceral adipose index; VAT, visceral adipose tissue; IMAT, intermuscular adipose tissue; SAI, subcutaneous adipose index; SM, skeletal muscle; ESR, Erythrocyte sedimentation rate; HR: Hazards Ratio; CI, confidence interval; CTE, CT enterography. Complete lists of all variables analyzed (including non-significant covariates) are provided in Supplementary Material 5.

In the female cohort, significant univariate predictors included: increased SAT density at L3–L4 level (*p* = 0.005, *p* = 0.012), VAT density at L4 level (*p* < 0.001), decreased SAI at L4-L5 level (*p* = 0.045, *p* = 0.031), and VAI at L5 level (*p* = 0.008) at Time 1; and increased SMI at L3 level (*p* = 0.001), SAT density at L3–L5 level (*p* = 0.015, *p* = 0.013), VAT density at L3–L5 level (*p* = 0.034, *p* = 0.018, *p* = 0.002), and decreased VAI at L3 level (*p* < 0.001) at Time 2. Following covariate adjustment, only VAI at L3 level at Time 2 (HR 0.949; 95% CI 0.919–0.980; *p* = 0.002), delta SMI at L3 level (HR 21.421; 95% CI 2.471–185.682; *p* = 0.005), ESR (HR 1.221; 95% CI 1.074–1.388; *p* = 0.002), albumin (HR 0.915; 95% CI 0.866–0.967; *p* = 0.002), and length of the abnormal small bowel segment (HR 1.068; 95% CI 1.025–1.112; *p* = 0.002) retained independent prognostic significance (Table 5). Owing to space limitations, Tables 4, 5 present statistically significant indicators (*p* < 0.05) from the univariate analysis for predicting high risk in CD patients. Complete results of both univariate and multivariate analyzes are provided in Supplementary Material 6.

**TABLE 5:** Multivariate survival analysis results of Crohn’s disease patients in female.

Parameters		Univariate analysis			Multivariate analysis		
		β	*P*-value	HR (95% CI)	β	*P*-value	HR (95% CI)
Time 1	L3 SAT density	0.040	0.005	1.041 (1.012–1.070)			
	L4 SAI	−0.027	0.045	0.973 (0.947–0.999)			
	L4 SAT density	0.044	0.012	1.045 (1.010–1.082)			
	L4 VAT density	0.096	<0.001	1.100 (1.044–1.159)			
	L5 SAI	−0.030	0.031	0.970 (0.944–0.997)			
	L5 VAI	−0.046	0.008	0.955 (0.923–0.988)			
	L5 VAT density	0.068	0.009	1.070 (1.017–1.126)			
Time 2	L3 VAI	−0.046	<0.001	0.955 (0.931–0.980)	−0.052	0.002	0.949 (0.919–0.980)
	L3 SMI	0.059	0.001	1.061 (1.023–1.101)			
	L3 SAT density	0.036	0.015	1.036 (1.007–1.067)			
	L3 VAT density	0.056	0.034	1.057 (1.004–1.113)			
	L4 SAT density	0.046	0.013	1.047 (1.010–1.086)			
	L4 VAT density	0.061	0.018	1.063 (1.010–1.117)			
	L5 VTR	−4.315	0.083	0.013 (0.000–1.744)			
	L5 SAT density	0.030	0.056	1.031 (0.999–1.063)			
	L5 VAT density	0.099	0.002	1.104 (1.037–1.175)			
Delta	L3 SMI	1.507	0.016	4.513 (1.319–15.442)	3.064	0.005	21.421 (2.471–185.682)
	L4 SAI	0.163	0.013	1.177 (1.035–1.339)			
	L5 VAI	0.124	0.023	1.132 (1.017–1.259)			
Clinical	Platelet	0.011	0.033	1.012 (1.001–1.022)			
	ESR	0.183	<0.001	1.200 (1.090–1.321)	0.199	0.002	1.221 (1.074–1.388)
	Calprotectin	0.003	0.007	1.003 (1.001–1.005)			
	Albumin	−0.101	<0.001	0.904 (0.862–0.947)	−0.089	0.002	0.915 (0.866–0.967)
CTE	Length	0.055	0.001	1.057 (1.021–1.093)	0.065	0.002	1.068 (1.025–1.112)

IMAI, intermuscular adipose index; SAT, subcutaneous adipose tissue; SMI, skeletal muscle index; VAI, visceral adipose index; VAT, visceral adipose tissue; IMAT, intermuscular adipose tissue; SAI, subcutaneous adipose index; SM, skeletal muscle; ESR, Erythrocyte sedimentation rate; HR: Hazards Ratio; CI, confidence interval; CTE, CT enterography.

### 3.4 Development and validation of the nomogram

**FIGURE 6:**
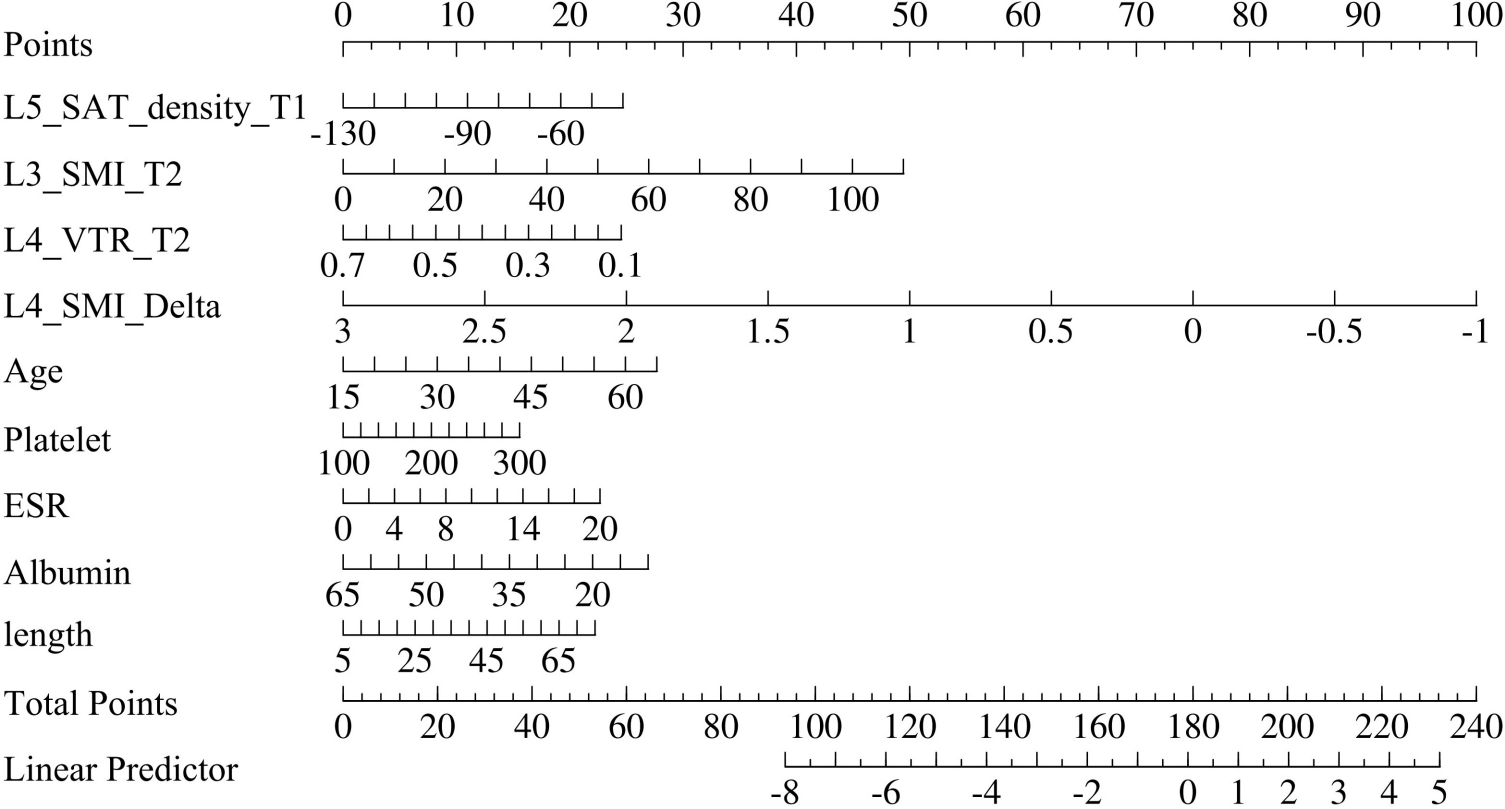
Nomogram for predicting the probability of high-risk patients with Crohn’s disease. T1, Time 1; T2, Time 2; SAT, subcutaneous adipose tissue; SMI, skeletal muscle index; VTR, VAT/total adipose tissue index; ESR, erythrocyte sedimentation rate.

The nine predictive factors identified in the previous section were incorporated into the analysis to construct a nomogram (Figure 6). In the training cohort, the AUC of ROC curve for nomogram at 12 months, 18 months, and 21 months were 0.877 (95% CI: 0.812–0.943), 0.878 (95% CI: 0.830–0.925), and 0.893 (95% CI: 0.845–0.941). In the test cohort, the predicted AUC values for 12-month, 18-month, and 21-months were 0.765 (95% CI: 0.601–0.929), 0.779 (95% CI: 0.669–0.888), and 0.799 (95% CI: 0.691–0.906), respectively (Supplementary Figure 4). The clinical practicability of the nomogram was evaluated by Decision Curve Analysis (DCA), and DCA demonstrated a net benefit with reasonable threshold probabilities (Supplementary Figure 5). The calibration curve of the training and verification cohort (1,000 bootstrap iterations) shows stable and extensible prediction performance (Supplementary Figure 6).

## 4 Discussion

To our knowledge, this is the first study to comprehensively investigate the relationship between dynamic body composition parameters obtained from CTE scans and the prognosis of patients with small bowel stenosis in CD. Our findings demonstrate that dynamic changes in body composition, quantified as delta parameters, provide critical prognostic insights beyond baseline measurements. Specifically, a decrease in L4 SMI emerged as a potent independent predictor of surgical necessity, suggesting that mitigating sarcopenia progression through nutritional or physical interventions may significantly reduce surgical risk. Sex-specific delta parameters, such as decreasing L3 VAT density in males and increasing L4 SAI in females, offer personalized biomarkers for risk stratification. Monitoring these trajectories using routine CTE could enable earlier, targeted interventions for high-risk patients.

While body composition changes may partly reflect disease refractoriness, their predictive value persisted after rigorous adjustment for baseline severity and dynamic therapy changes. This supports dynamic body composition as a surrogate of metabolic resilience beyond conventional biomarkers. After the treatment of small bowel stenosis in CD patients, muscle and fat parameters exhibited heterogeneous changes across different anatomical sites. In male and female patients, the SMI at the L3 level significantly decreased, indicating sarcopenia of the trunk muscles. Muscle loss in CD is multifactorial, involving chronic inflammation, malnutrition, and reduced physical activity (27–29), which may be related to treatment-induced muscle catabolism and malnutrition (30, 31). Notably, our study observed sex-specific differences in delta SMI, with males exhibiting more pronounced reductions at L3–L4 levels. This may reflect androgen-dependent muscle maintenance or differential inflammatory responses (32), underscoring the need for gender-stratified risk assessment in CD. Males exhibited significant VAT density reductions at L4–L5 but not at L3, aligning with sex-specific fat redistribution patterns in CD progression. While body composition changes may partly reflect disease refractoriness, their predictive value persisted after rigorous adjustment for baseline severity and dynamic therapy changes. This supports dynamic body composition as a surrogate of metabolic resilience beyond conventional biomarkers. This underscores the importance of longitudinal over single-timepoint assessment. The absence of sex-based anatomical differences reinforces that dynamic body composition changes, not static structural factors, drove surgical risk prediction in our models. Low SMI and high VAT density at T1 signify inflammation-driven catabolic wasting, a malnutrition phenotype distinct from isolated calorie deficiency. This metabolic derangement contributes to adverse outcomes independent of BMI. The robust protective effect of increasing SMI aligns with mechanistic studies linking sarcopenia to intestinal fibrosis. Clinically, this underscores that nutritional or physical interventions preserving muscle mass may delay surgical intervention, even in high-risk stricturing phenotypes.

Regarding fat parameters, in both the male subgroups, the VAT density at the L4 and L5 levels significantly decreased, which may be related to local metabolic changes after treatment (33). However, the SAT parameters showed no significant change, indicating that the subcutaneous tissue remained stable. The short treatment may not have been sufficient to cause noticeable changes. Future studies with more frequent intervals should validate these findings and explore the underlying mechanisms driving these changes. This finding aligns with the broader literature on gender differences in body composition and its impact on health outcomes (11). For example, studies have shown that males tend to have higher visceral adiposity, which is associated with increased inflammation and metabolic risk, while females have relatively higher subcutaneous adiposity, which may have a more protective role (34, 35). In male, the increase in IMAI at L3 level may indicate reduced local fat infiltration following the alleviation of inflammation. The muscle fat infiltration to surgery-requiring progression predictor was not yet fully understood. Several hypotheses exist, with the most prominent ones focusing on the relationship between myosteatosis, systemic inflammation, and cachexia (36, 37). The association between VAT density at L3 level and surgical risk is particularly intriguing. Higher VAT radiodensity, indicative of lipid peroxidation and inflammation, has been linked to systemic metabolic dysfunction in obesity-related disorders (38, 39). In CD, VAT accumulation may promote intestinal fibrosis through adipokine secretion and macrophage infiltration (40).

Prior studies have explored baseline body composition in CD, reporting associations between visceral adiposity and postoperative complications (13, 31). Van Der Sloot et al. found that visceral adiposity was associated with an increased risk of complications in CD (16). However, these studies primarily focused on static measurements of body composition at a single time point. In contrast, our study evaluates the prognostic value of changes in body composition over time, providing a more dynamic and comprehensive assessment of disease progression. For instance, our finding that decreasing delta SMI predicts surgery suggests that dynamic changes in muscle tissue composition may better reflect disease activity. These findings extend the work of Thiberge et al., who reported that low subcutaneous adiposity predicts adverse outcomes in CD (18), by emphasizing the importance of temporal dynamics. Current guidelines rely on static imaging parameters like stricture length and luminal diameter (41), which fail to account for metabolic changes preceding clinical deterioration. The nomogram in this study, combining delta SMI, SAT density, and clinical parameters, offers improved discrimination, potentially enabling earlier surgical intervention for high-risk patients. Notably, 52.2% of patients presented with perianal lesions alongside small bowel strictures, reflecting a phenotype associated with heightened systemic inflammation and worse prognoses. This dual pathology may exacerbate muscle catabolism and visceral adipose dysfunction, further amplifying surgical risk.

Although disease duration was marginally longer in the high-risk group, it did not independently predict surgical risk or significantly correlate with longitudinal body composition changes in multivariate models. This suggests that while chronicity may contribute to tissue remodeling, it does not confound the prognostic value of dynamic CT-based body composition metrics. In our cohort, ileocolonic involvement predominated, contrasting with Western cohorts reporting higher isolated ileal rates. This aligns with recent Asian studies attributing L3 predominance to contiguous inflammation patterns from terminal ileum to cecum (42). Critically, disease localization did not independently influence surgical risk prediction in our models. We acknowledge that while regional criteria enhance internal validity, they may limit direct comparability with Western cohorts. This methodological distinction underscores the need for geographic contextualization when comparing phenotypic distributions.

Our study has some limitations. Firstly, the included CD patients were all Chinese, and this is a single-center retrospective study. Second, the 6–12 months follow-up period may not capture long-term outcomes, necessitating validation in prospective studies. Third, while we used advanced imaging techniques to measure body composition, the accuracy of these measurements could be further improved with emerging technologies such as artificial intelligence-based segmentation tools. Fourth, despite subgroup analyzes, residual confounding by treatment heterogeneity cannot be fully excluded; future prospective studies should standardize therapeutic protocols. While CTE enables high-resolution body composition and stricture evaluation, cumulative radiation exposure remains a limitation. We mitigated this via dose-optimization protocols and clinically justified intervals. Future studies should validate our findings using low-dose CT or emerging non-ionizing modalities (e.g., MR-based fat quantification) where feasible. HR should be interpreted as prognostic associations; causal inference requires prospective intervention trials targeting body composition.

This study establishes that dynamic body composition parameters, particularly delta SMI and adipose tissue parameters, serve as valuable imaging biomarkers for predicting surgical necessity in CD patients with small bowel stenosis. These readily obtainable metrics may be integrated into routine clinical practice to facilitate personalized risk stratification and therapeutic decision-making.

## Data Availability

The original contributions presented in the study are included in the article/Supplementary material, further inquiries can be directed to the corresponding authors.
