# Supplementary material for: Dynamic CT-based body composition analysis predicts surgical risk in Crohn’s disease with small bowel stenosis: a retrospective cohort study
Source: Front Med (Lausanne). 2025 Sep 18;12:1642851. doi: 10.3389/fmed.2025.1642851 (PMC12488570; doi:10.3389/fmed.2025.1642851)
Supplement: Supplementary file 1 [file Data_Sheet_1.pdf]

Supplementary Material

1 Supplementary Material 1: The classification of small bowel Crohn's disease.

Figure S1

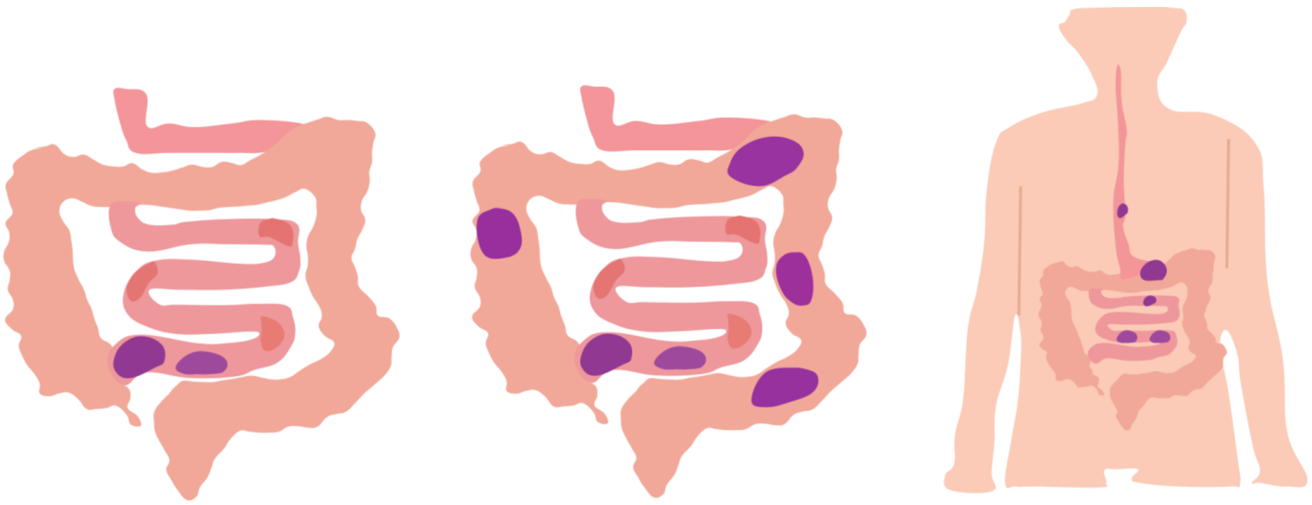

L1 L3 L4

Note: The classification of small bowel Crohn's disease (CD) by location and disease behavior is defined per the Consensus Statement on Endoscopic Diagnosis and Treatment for Small Bowel Crohn's Disease, wherein: L1 denotes disease confined to the terminal ileum; L3 indicates involvement of both terminal ileum and colon; L4 denotes involvement of the upper gastrointestinal tract.

References: DOI： 10.3760/cma.j.cn101480-20250103-00004

2 Supplementary Material 2: Abdominal body composition parameter extraction methods.

Three slices were evenly selected at the level of the third to fifth lumbar vertebrae for each patient, and a total of 9 slices were used for subsequent analysis (Figure S1-a). Two radiologists used the semi-automatic method of SlicerOmatic to outline the skeletal muscle (SM), subcutaneous adipose tissue (SAT), visceral adipose tissue (VAT), and intermuscular adipose tissue (IMAT) in the above-mentioned images. The main steps were to outline the SM by setting a threshold of -29 to +150 HU, to outline the SAT and IMAT by setting a threshold of -190 to -30 HU, and to outline the VAT by setting a threshold of -150 to -50 HU. Thresholds and segmentation protocols were applied per established standards in body composition literature, as referenced. The correction was carried out by another radiologist, mainly including the inspection and correction of the preliminary results. The area and mean attenuation of SM, SAT, VAT, and IMAT were quantified. Normalized indices were obtained by dividing the area of each component by the square of the vertebral height (L1-L5) (Figure S1-b).

Figure S2

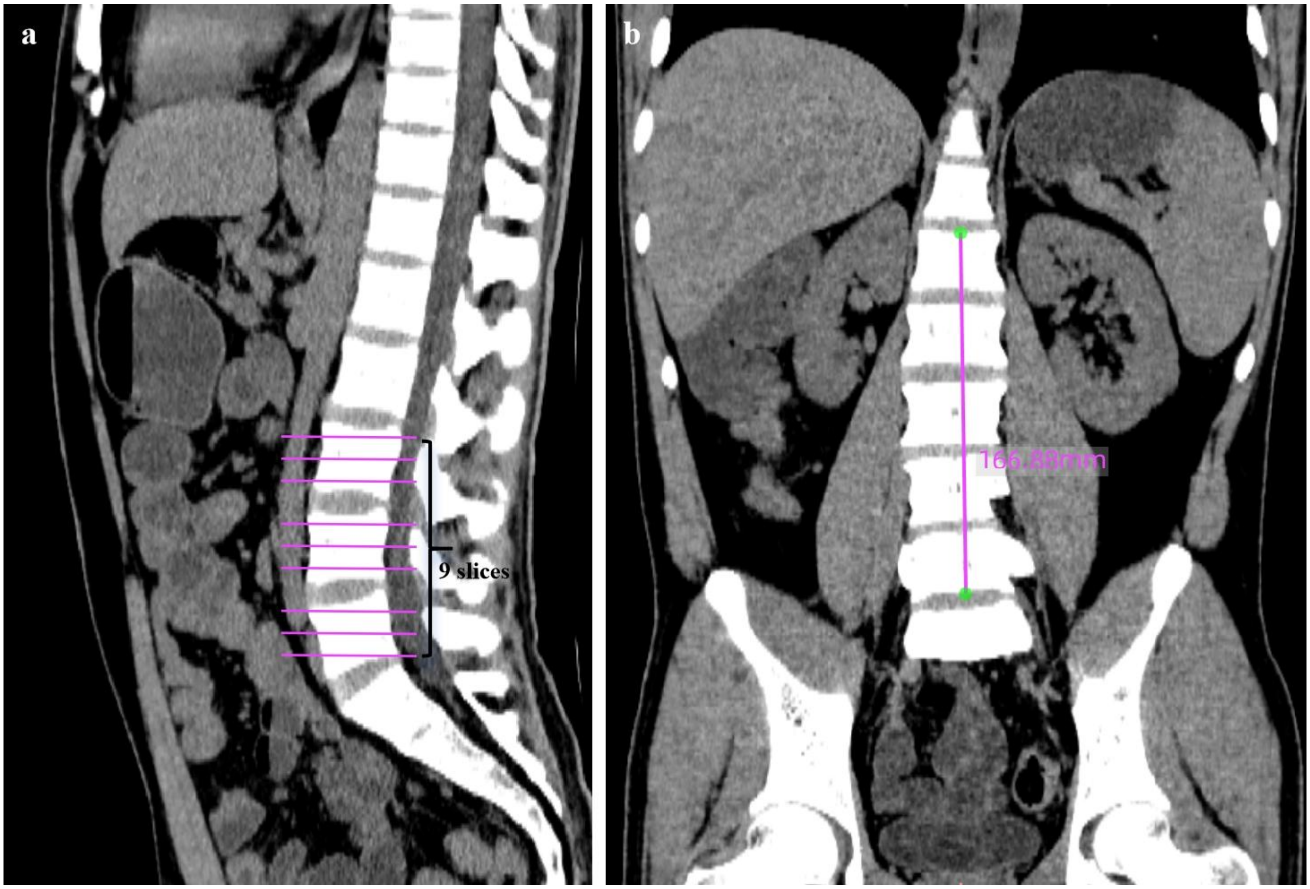

Note: a) The selection of slice to be analyzed; b) A coronal CT image shows the measurement of vertebral height (L1-L5).

Figure S3

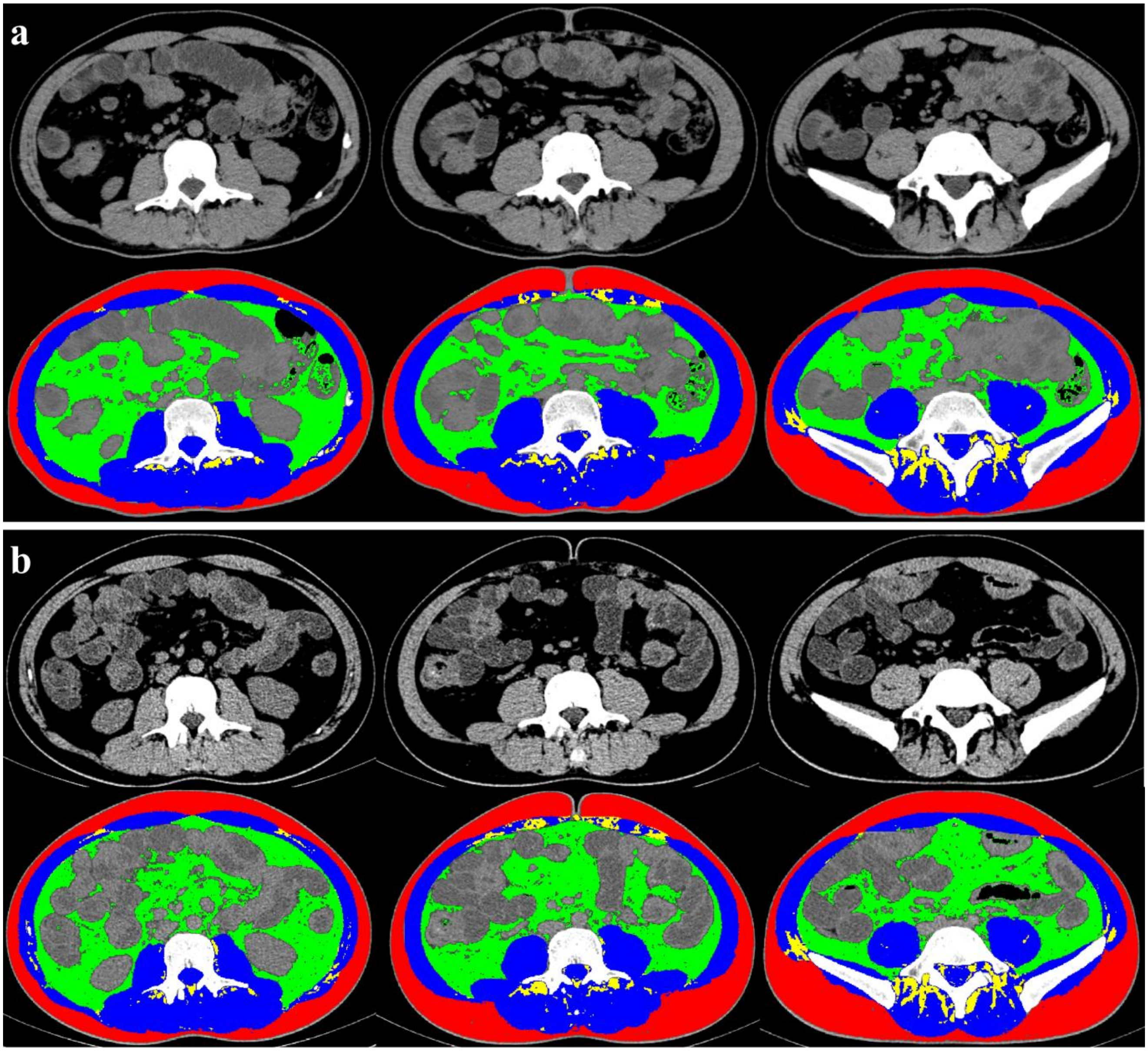

Note: a) Schematic diagram of the delineation of abdominal body composition parameter by computed tomography enterography (CTE) for the patient at the first time; b) Schematic diagram of the delineation of abdominal body composition parameter by CTE for the patient at the second time. Blue represents the skeletal muscle (SM), red represents the subcutaneous adipose tissue (SAT), green represents the visceral adipose tissue (VAT), and the yellow represents the intermuscular adipose tissue (IMAT).

3 **Supplementary Material 3:** Baseline characteristics of Crohn's disease patients.

Table S1. Baseline characteristics of Crohn's disease patients in male and female cohorts.

Characteristics	Male (n=304)	Female (n=81)	*p* value
Age (year)	33.80±10.65	33.27±9.72	0.689
Disease duration (year)	2.67±0.71	2.69±0.85	0.878
Leukocyte (10^9/L)	7.18 ± 2.18	7.84 ± 2.60	0.038
Hemoglobin (g/L)	119.63 ± 21.71	118.93 ± 22.68	0.799
Platelet (10^9/L)	217.85 ± 51.11	221.23 ± 52.35	0.598
Hematocrit (%)	39.00 ± 5.36	38.85 ± 5.93	0.822
CRP (mg/L)	38.24 ± 36.04	49.01 ± 49.07	0.068
ESR (mm/h)	8.85 ± 5.09	8.46 ± 4.97	0.536
FC (μg/g)	256.25 ± 172.43	279.88 ± 175.43	0.276
Albumin (g/L)	37.80 ± 8.14	37.70 ± 8.23	0.922
L1 (N, %)	37 (12.2%)	12 (14.8%)	0.798
L3 (N, %)	267 (87.8%)	69 (85.2%)	
#L4 (N, %)	21 (6.9%)	5 (6.2%)	
P (N, %)	157 (51.6%)	44 (54.3%)	0.668
Immunomodulators (N, %)	82 (27.0%)	19 (23.4%)	
Biologics (N, %)	177 (58.2%)	48 (59.3%)	0.752
Combination (N, %)	45 (14.8%)	14 (17.3%)	
CTE features	-	-	-
Length (mm)	35.82 ± 17.37	35.44 ± 15.47	0.860
Diameter (mm)	8.10 ± 3.57	7.38 ± 2.79	0.052
Proximal (mm)	31.96 ± 16.28	31.04 ± 12.16	0.637
Thickness (mm)	7.47 ± 2.19	7.56 ± 2.88	0.758
Comb sign (N, %)	184 (60.5%)	52 (64.2%)	0.547
Lymph nodes (N, %)	253 (83.2%)	67 (82.7%)	0.914
Stricture with inflammation (N, %)	216 (71.1%)	58 (71.6%)	0.752
Stricture without inflammation (N, %)	88 (28.9%)	23 (28.4%)	
Single-focal strictures (N, %)	218 (71.7%)	59 (72.8%)	
Multifocal strictures (N, %)	86 (28.3%)	22 (27.2%)	

Note: CRP, C-reactive protein; ESR, Erythrocyte sedimentation rate; FC, Fecal calprotectin; L1, ileal; L3, ileocolonic; L4, upper digestive tract; # was coexist with other disease location. P, perianal disease; CTE, CT enterography.

Table S2. Baseline characteristics of Crohn's disease patients in training and test cohorts.

Characteristics	Training (n=269)	Test (n=116)	*p* value
Age (year)	33.29±10.06	34.60±11.19	0.178
Male (%)	210 (78.0%)	94 (81.0%)	0.603
Female (%)	59 (22.0%)	22 (19.0%)	
Disease duration (year)	2.65±0.71	2.74±0.79	0.271
Leukocyte (10^9/L)	7.2±2.3	7.3±2.2	0.949
Hemoglobin (g/L)	119.4±21.5	119.5±22.8	0.946
Platelet (10^9/L)	219.8±51.7	215.6±50.5	0.461
Hematocrit (%)	38.8±5.4	39.2±5.4	0.555
CRP (mg/L)	40.7±38.2	40.0±41.8	0.875
ESR (mm/h)	8.9±5.1	8.4±4.9	0.390
FC (μg/g)	258.1±172.6	268.1±174.7	0.603
Albumin (g/L)	37.6±8.0	38.1±8.4	0.594
L1 (N, %)	36 (13.3%)	13 (11.2%)	0.517
L3 (N, %)	232 (86.2%)	104 (89.6%)	
[#]L4 (N, %)	19 (7.0 %)	12 (10.3%)	
P (N, %)	146 (54.2%)	55 (47.4%)	0.216
Immunomodulators (N, %)	74 (27.5%)	27 (23.3%)	0.244
Biologics (N, %)	159 (59.1%)	66 (56.9%)	
Combination (N, %)	36 (13.4%)	23 (19.8%)	
CTE features	-	-	-
Length (mm)	35.0±15.9	37.2±19.0	0.258
Diameter (mm)	7.9±3.4	8.0±3.3	0.763
Proximal (mm)	35.0±15.9	37.2±19.0	0.415
Thickness (mm)	7.4±2.2	7.4±2.5	0.930
Comb sign (N, %)	158 (58.7%)	78 (67.2%)	0.116
Lymph nodes (N, %)	219 (81.4%)	101 (87.0%)	0.174
Stricture with inflammation (N, %)	192 (71.4%)	82 (70.7%)	0.892
Stricture without inflammation (N, %)	77 (28.6%)	34 (29.3%)	
Single-focal strictures (N, %)	194 (72.1%)	83 (71.6%)	0.909
Multifocal strictures (N, %)	75 (27.9%)	33 (28.4%)	

Note: CRP, C-reactive protein; ESR, Erythrocyte sedimentation rate; FC, Fecal calprotectin; L1, ileal; L3, ileocolonic; L4, upper digestive tract; [#] was coexist with other disease location. P, perianal disease; CTE, CT enterography.

4 Supplementary Material 4: Body composition parameters of the Crohn's disease patients in training and test cohorts.

Table S3: Body composition parameters of the Crohn's disease patients in training and test cohorts.

Variables		Time 1			Time 2		
		Training	Test	*p* value	Training	Test	*p* value
L3	IMAI (10^2 cm²/m²)	3.1±2.1	3.2±1.9	0.678	2.6±1.5	2.4±1.5	0.302
	IMAT density (HU)	-60.4±5.6	-60.6±6.1	0.766	-59.1±6.0	-60.0±6.2	0.086
	SAI (10^2 cm²/m²)	42.4±16.4	41.8±16.7	0.780	43.2±22.0	45.0±23.8	0.481
	SAT density (HU)	-92.2±14.4	-89.2±12.1	0.053	-91.1±13.4	-91.4±12.8	0.861
	SMI (10^2 cm²/m²)	61.8±13.7	64.4±13.3	0.085	67.6±14.7	68.3±13.7	0.662
	SM density (HU)	41.6±7.4	42.0±7.3	0.622	42.3±7.9	42.5±7.3	0.827
	VAI (10^2 cm²/m²)	40.1±17.7	41.7±19.1	0.440	37.8±22.7	40.0±21.6	0.376
	VAT density (HU)	-90.6±8.9	-90.2±9.1	0.692	-89.5±9.6	-89.6±9.5	0.918
	VSR	0.9±0.4	1.0±0.5	0.088	0.8±0.4	0.8±0.4	0.395
	VTR	0.4±0.1	0.4±0.1	0.947	0.4±0.1	0.4±0.1	0.681
L4	IMAI (10^2 cm²/m²)	2.8±1.7	2.9±1.6	0.488	3.1±1.8	2.8±1.5	0.192
	IMAT density (HU)	-60.6±5.7	-60.6±5.7	0.910	-60.1±6.1	-59.6±6.5	0.475
	SAI (10^2 cm²/m²)	43.3±14.3	43.4±12.4	0.929	43.6±18.6	42.8±18.0	0.694
	SAT density (HU)	-92.7±12.4	-92.2±13.1	0.702	-93.2±13.6	-92.1±12.8	0.473
	SMI (10^2 cm²/m²)	62.7±12.1	63.1±11.4	0.756	66.3±13.7	66.0±13.6	0.868
	SM density (HU)	43.4±7.2	41.9±7.3	0.078	42.6±7.2	43.1±7.6	0.573
	VAI (10^2 cm²/m²)	40.9±13.3	41.0±13.4	0.988	39.2±20.8	38.8±17.3	0.853
	VAT density (HU)	-91.7±8.4	-91.4±8.7	0.798	-89.6±8.8	-89.8±9.7	0.876
	VSR	0.7±0.3	0.7±0.3	0.745	0.7±0.3	0.7±0.3	0.813
	VTR	0.3±0.1	0.4±0.1	0.303	0.3±0.1	0.3±0.1	0.775
L5	IMAI (10^2 cm²/m²)	4.6±2.2	4.6±1.9	0.715	5.0±2.5	4.4±2.4	0.022
	IMAT density (HU)	-65.9±7.8	-64.9±7.8	0.252	-66.1±7.3	-64.8±7.8	0.125
	SAI (10^2 cm²/m²)	39.7±12.9	39.5±12.6	0.910	42.4±16.3	43.0±15.3	0.750
	SAT density (HU)	-91.8±13.7	-91.1±13.4	0.659	-91.0±13.5	-88.9±13.2	0.153
	SMI (10^2 cm²/m²)	61.4±11.1	62.8±10.0	0.243	61.9±12.0	63.2±11.5	0.344
	SM density (HU)	41.0±7.1	41.7±7.5	0.352	42.2±6.6	43.7±7.9	0.057
	VAI (10^2 cm²/m²)	35.0±14.6	34.2±14.9	0.603	34.0±15.2	37.7±15.3	0.028
	VAT density (HU)	-90.6±8.3	-91.8±9.2	0.246	-88.9±7.9	-89.3±7.5	0.695
	VSR	0.6±0.3	0.6±0.3	0.418	0.6±0.3	0.6±0.2	0.987
	VTR	0.3±0.1	0.3±0.1	0.910	0.3±0.1	0.3±0.1	0.687

Note: IMAI, intermuscular adipose index; IMAT, intermuscular adipose tissue; SAI, subcutaneous adipose index; SAT, subcutaneous adipose tissue; SMI, skeletal muscle index; SM, skeletal muscle; VAI, visceral adipose index; VAT, visceral adipose tissue; VAR, VAT/SAT ratio; VTR, VAT/total adipose tissue index.

5 Supplementary Material 5: Univariate and multivariate survival analysis for predicting high risk patients (Training cohort).

Table S4. Univariate and multivariate survival analysis for predicting high risk patients (Time 1).

Variables	Univariate analysis			Multivariate analysis		
	β	*p* value	HR (95% CI)	β	*p* value	HR (95% CI)
L3 SAI	-0.007	0.199	0.993 (0.982-1.004)			
L3 VAI	-0.004	0.421	0.996 (0.985-1.006)			
L3 SMI	-0.003	0.699	0.997 (0.982-1.012)			
L3 IMAI	0.037	0.641	1.038 (0.888-1.212)			
L3 VSR	0.219	0.434	1.245 (0.720-2.152)			
L3 VTR	-1.584	0.128	0.205 (0.027-1.577)			
L3 SAT density	0.030	<0.001	1.030 (1.013-1.048)			
L3 VAT density	-0.002	0.854	0.998 (0.972-1.024)			
L3 SM density	-0.035	0.022	0.965 (0.936-0.995)			
L3 IMAT density	-0.011	0.621	0.989 (0.949-1.032)			
L4 SAI	-0.018	0.014	0.982 (0.969-0.996)	-0.017	0.011	0.983 (0.970-0.996)
L4 VAI	0.000	0.969	1.000 (0.989-1.012)			
L4 SMI	0.021	0.009	1.021 (1.005-1.037)	0.027	0.002	1.028 (1.010-1.045)
L4 IMAI	0.019	0.760	1.019 (0.902-1.152)			
L4 VSR	0.249	0.449	1.282 (0.674-2.441)			
L4 VTR	-0.542	0.639	0.581 (0.060-5.604)			
L4 SAT density	0.030	<0.001	1.031 (1.014-1.048)	0.027	0.001	1.027 (1.011-1.044)
L4 VAT density	0.034	0.018	1.035 (1.006-1.064)			
L4 SM density	-0.010	0.541	0.990 (0.958-1.023)			
L4 IMAT density	0.014	0.467	1.014 (0.976-1.054)			
L5 SAI	-0.018	0.012	0.982 (0.968-0.996)			
L5 VAI	-0.022	0.012	0.979 (0.962-0.995)			
L5 SMI	0.013	0.233	1.013 (0.992-1.034)			
L5 IMAI	0.033	0.468	1.034 (0.946-1.130)			
L5 VSR	0.474	0.199	1.607 (0.779-3.314)			
L5 VTR	0.563	0.679	1.756 (0.122-25.329)			
L5 SAT density	0.021	0.021	1.021 (1.003-1.039)			
L5 VAT density	0.021	0.202	1.021 (0.989-1.054)			
L5 SM density	-0.049	0.008	0.952 (0.918-0.987)	-0.057	0.003	0.944 (0.909-0.981)
L5 IMAT density	0.010	0.566	1.010 (0.977-1.043)			

Note: IMAI, intermuscular adipose index; IMAT, intermuscular adipose tissue; SAI, subcutaneous adipose index; SAT, subcutaneous adipose tissue; SMI, skeletal muscle index; SM, skeletal muscle; VAI, visceral adipose index; VAT, visceral adipose tissue; VAR, VAT/SAT ratio; VTR, VAT/total adipose tissue index; HR: Hazards Ratio; CI, confidence interval.

Table S5. Univariate and multivariate survival analysis for predicting high risk patients (Time 2).

Variables	Univariate analysis			Multivariate analysis		
	β	*p* value	HR (95% CI)	β	*p* value	HR (95% CI)
L3 SAI	-0.009	0.235	0.991 (0.975-1.006)			
L3 VAI	-0.020	0.005	0.980 (0.966-0.994)			
L3 SMI	0.057	<0.001	1.059 (1.039-1.080)	0.053	<0.001	1.054 (1.034-1.075)
L3 IMAI	-0.044	0.451	0.957 (0.853-1.073)			
L3 VSR	-0.329	0.202	0.719 (0.434-1.193)			
L3 VTR	-1.654	0.106	0.191 (0.026-1.424)			
L3 SAT density	0.020	0.016	1.020 (1.004-1.037)			
L3 VAT density	0.036	0.006	1.036 (1.010-1.063)			
L3 SM density	-0.013	0.383	0.987 (0.957-1.017)			
L3 IMAT density	0.025	0.238	1.025 (0.984-1.069)			
L4 SAI	0.003	0.741	1.003 (0.987-1.019)			
L4 VAI	-0.019	0.042	0.982 (0.964-0.999)			
L4 SMI	-0.014	0.146	0.986 (0.967-1.005)			
L4 IMAI	-0.133	0.076	0.875 (0.755-1.014)			
L4 VSR	0.102	0.767	1.107 (0.564-2.175)			
L4 VTR	-2.550	0.036	0.078 (0.007-0.842)			
L4 SAT density	0.021	0.023	1.022 (1.003-1.041)			
L4 VAT density	0.054	<0.001	1.055 (1.027-1.084)			
L4 SM density	-0.011	0.492	0.989 (0.957-1.021)			
L4 IMAT density	0.075	<0.001	1.078 (1.034-1.124)	0.055	0.015	1.057 (1.011-1.105)
L5 SAI	-0.008	0.396	0.992 (0.975-1.010)			
L5 VAI	-0.018	0.036	0.983 (0.967-0.999)			
L5 SMI	-0.011	0.311	0.989 (0.969-1.010)			
L5 IMAI	-0.008	0.876	0.992 (0.893-1.101)			
L5 VSR	-0.133	0.718	0.876 (0.426-1.799)			
L5 VTR	-3.015	0.017	0.049 (0.004-0.582)			
L5 SAT density	0.020	0.024	1.020 (1.003-1.038)			
L5 VAT density	0.067	<0.001	1.069 (1.038-1.101)	0.052	<0.001	1.053 (1.026-1.082)
L5 SM density	-0.024	0.146	0.976 (0.945-1.008)			
L5 IMAT density	0.033	0.043	1.034 (1.001-1.067)			

Note: IMAI, intermuscular adipose index; IMAT, intermuscular adipose tissue; SAI, subcutaneous adipose index; SAT, subcutaneous adipose tissue; SMI, skeletal muscle index; SM, skeletal muscle; VAI, visceral adipose index; VAT, visceral adipose tissue; VAR, VAT/SAT ratio; VTR, VAT/total adipose tissue index; HR: Hazards Ratio; CI, confidence interval.

Table S6. Univariate and multivariate survival analysis for predicting high risk patients (Time 1, Time 2, delta).

Variables	Univariate analysis			Multivariate analysis		
	β	*p* value	HR (95% CI)	β	*p* value	HR (95% CI)
Time 1	-	-	-	-	-	-
L3 SAI	-0.007	0.199	0.993 (0.982-1.004)			
L3 VAI	-0.004	0.421	0.996 (0.985-1.006)			
L3 SMI	-0.003	0.699	0.997 (0.982-1.012)			
L3 IMAI	0.037	0.641	1.038 (0.888-1.212)			
L3 VSR	0.219	0.434	1.245 (0.720-2.152)			
L3 VTR	-1.584	0.128	0.205 (0.027-1.577)			
L3 SAT density	0.030	<0.001	1.030 (1.013-1.048)			
L3 VAT density	-0.002	0.854	0.998 (0.972-1.024)			
L3 SM density	-0.035	0.022	0.965 (0.936-0.995)	-0.034	0.031	0.966 (0.936-0.997)
L3 IMAT density	-0.011	0.621	0.989 (0.949-1.032)			
L4 SAI	-0.018	0.014	0.982 (0.969-0.996)			
L4 VAI	0.000	0.969	1.000 (0.989-1.012)			
L4 SMI	0.021	0.009	1.021 (1.005-1.037)			
L4 IMAI	0.019	0.760	1.019 (0.902-1.152)			
L4 VSR	0.249	0.449	1.282 (0.674-2.441)			
L4 VTR	-0.542	0.639	0.581 (0.060-5.604)			
L4 SAT density	0.030	<0.001	1.031 (1.014-1.048)			
L4 VAT density	0.034	0.018	1.035 (1.006-1.064)			
L4 SM density	-0.010	0.541	0.990 (0.958-1.023)			
L4 IMAT density	0.014	0.467	1.014 (0.976-1.054)			
L5 SAI	-0.018	0.012	0.982 (0.968-0.996)			
L5 VAI	-0.022	0.012	0.979 (0.962-0.995)			
L5 SMI	0.013	0.233	1.013 (0.992-1.034)			
L5 IMAI	0.033	0.468	1.034 (0.946-1.130)			
L5 VSR	0.474	0.199	1.607 (0.779-3.314)			
L5 VTR	0.563	0.679	1.756 (0.122-25.329)			
L5 SAT density	0.021	0.021	1.021 (1.003-1.039)			
L5 VAT density	0.021	0.202	1.021 (0.989-1.054)			
L5 SM density	-0.049	0.008	0.952 (0.918-0.987)	-0.052	0.012	0.949 (0.912-0.989)
L5 IMAT density	0.010	0.566	1.010 (0.977-1.043)			
Time 2	-	-	-	-	-	-
L3 SAI	-0.009	0.235	0.991 (0.975-1.006)			
L3 VAI	-0.020	0.005	0.980 (0.966-0.994)			
L3 SMI	0.057	<0.001	1.059 (1.039-1.080)	0.051	<0.001	1.053 (1.032-1.074)
L3 IMAI	-0.044	0.451	0.957 (0.853-1.073)			
L3 VSR	-0.329	0.202	0.719 (0.434-1.193)			
L3 VTR	-1.654	0.106	0.191 (0.026-1.424)			
L3 SAT density	0.020	0.016	1.020 (1.004-1.037)			
L3 VAT density	0.036	0.006	1.036 (1.010-1.063)			
L3 SM density	-0.013	0.383	0.987 (0.957-1.017)			
L3 IMAT density	0.025	0.238	1.025 (0.984-1.069)			
L4 SAI	0.003	0.741	1.003 (0.987-1.019)			
L4 VAI	-0.019	0.042	0.982 (0.964-0.999)			

Variables	Univariate analysis			Multivariate analysis		
	β	*p* value	HR (95% CI)	β	*p* value	HR (95% CI)
L4 SMI	-0.014	0.146	0.986 (0.967-1.005)			
L4 IMAI	-0.133	0.076	0.875 (0.755-1.014)			
L4 VSR	0.102	0.767	1.107 (0.564-2.175)			
L4 VTR	-2.550	0.036	0.078 (0.007-0.842)			
L4 SAT density	0.021	0.023	1.022 (1.003-1.041)			
L4 VAT density	0.054	<0.001	1.055 (1.027-1.084)			
L4 SM density	-0.011	0.492	0.989 (0.957-1.021)			
L4 IMAT density	0.075	<0.001	1.078 (1.034-1.124)	0.049	0.023	1.050 (1.007-1.096)
L5 SAI	-0.008	0.396	0.992 (0.975-1.010)			
L5 VAI	-0.018	0.036	0.983 (0.967-0.999)			
L5 SMI	-0.011	0.311	0.989 (0.969-1.010)			
L5 IMAI	-0.008	0.876	0.992 (0.893-1.101)			
L5 VSR	-0.133	0.718	0.876 (0.426-1.799)			
L5 VTR	-3.015	0.017	0.049 (0.004-0.582)	-2.374	0.035	0.093 (0.010-0.842)
L5 SAT density	0.020	0.024	1.020 (1.003-1.038)			
L5 VAT density	0.067	<0.001	1.069 (1.038-1.101)	0.058	<0.001	1.060 (1.031-1.089)
L5 SM density	-0.024	0.146	0.976 (0.945-1.008)			
L5 IMAT density	0.033	0.043	1.034 (1.001-1.067)			
Delta	-	-	-	-	-	-
L3 SAI	-0.054	0.448	0.947 (0.824-1.089)			
L3 VAI	-0.108	0.252	0.898 (0.747-1.079)			
L3 SMI	0.116	0.358	1.123 (0.877-1.440)			
L3 IMAI	-0.151	0.137	0.860 (0.705-1.049)			
L3 VSR	-0.129	0.139	0.879 (0.741-1.043)			
L3 VTR	-0.022	0.939	0.978 (0.561-1.706)			
L3 SAT density	0.483	0.337	1.621 (0.605-4.339)			
L3 VAT density	-1.627	0.075	0.197 (0.033-1.176)			
L3 SM density	0.378	0.444	1.460 (0.554-3.847)			
L3 IMAT density	-1.088	0.237	0.337 (0.055-2.049)			
L4 SAI	-0.006	0.772	0.994 (0.954-1.036)			
L4 VAI	0.021	0.663	1.021 (0.930-1.120)			
L4 SMI	-1.691	0.001	0.184 (0.067-0.506)	-1.375	0.011	0.253 (0.087-0.733)
L4 IMAI	-0.123	0.238	0.884 (0.721-1.085)			
L4 VSR	-0.143	0.210	0.867 (0.693-1.084)			
L4 VTR	-0.427	0.184	0.653 (0.348-1.225)			
L4 SAT density	0.720	0.193	2.054 (0.694-6.080)			
L4 VAT density	-0.946	0.334	0.388 (0.057-2.649)			
L4 SM density	-0.248	0.644	0.781 (0.273-2.233)			
L4 IMAT density	-1.807	0.050	0.164 (0.027-1.002)			
L5 SAI	0.001	0.980	1.001 (0.912-1.099)			
L5 VAI	0.066	0.505	1.068 (0.880-1.296)			
L5 SMI	-0.651	0.158	0.522 (0.211-1.288)			
L5 IMAI	-0.007	0.667	0.993 (0.962-1.025)			
L5 VSR	-0.353	0.057	0.702 (0.488-1.011)			
L5 VTR	-0.864	0.020	0.422 (0.204-0.873)			

Variables	Univariate analysis			Multivariate analysis		
	β	*p* value	HR (95% CI)	β	*p* value	HR (95% CI)
L5 SAT density	0.049	0.935	1.050 (0.319-3.454)			
L5 VAT density	-2.645	0.013	0.071 (0.009-0.574)			
L5 SM density	0.459	0.464	1.582 (0.463-5.409)			
L5 IMAT density	-0.896	0.306	0.408 (0.073-2.271)			

Note: IMAI, intermuscular adipose index; IMAT, intermuscular adipose tissue; SAI, subcutaneous adipose index; SAT, subcutaneous adipose tissue; SMI, skeletal muscle index; SM, skeletal muscle; VAI, visceral adipose index; VAT, visceral adipose tissue; VAR, VAT/SAT ratio; VTR, VAT/total adipose tissue index; HR: Hazards Ratio; CI, confidence interval.

Table S7. Univariate and multivariate survival analysis for predicting high risk patients (Time 1, Time 2, delta, clinical and CTE imaging parameters).

Variables	Univariate analysis			Multivariate analysis		
	β	*p* value	HR (95% CI)	β	*p* value	HR (95% CI)
Time 1	-	-	-	-	-	-
L3 SAI	-0.007	0.199	0.993 (0.982-1.004)			
L3 VAI	-0.004	0.421	0.996 (0.985-1.006)			
L3 SMI	-0.003	0.699	0.997 (0.982-1.012)			
L3 IMAI	0.037	0.641	1.038 (0.888-1.212)			
L3 VSR	0.219	0.434	1.245 (0.720-2.152)			
L3 VTR	-1.584	0.128	0.205 (0.027-1.577)			
L3 SAT density	0.030	<0.001	1.030 (1.013-1.048)			
L3 VAT density	-0.002	0.854	0.998 (0.972-1.024)			
L3 SM density	-0.035	0.022	0.965 (0.936-0.995)			
L3 IMAT density	-0.011	0.621	0.989 (0.949-1.032)			
L4 SAI	-0.018	0.014	0.982 (0.969-0.996)			
L4 VAI	0.000	0.969	1.000 (0.989-1.012)			
L4 SMI	0.021	0.009	1.021 (1.005-1.037)			
L4 IMAI	0.019	0.760	1.019 (0.902-1.152)			
L4 VSR	0.249	0.449	1.282 (0.674-2.441)			
L4 VTR	-0.542	0.639	0.581 (0.060-5.604)			
L4 SAT density	0.030	<0.001	1.031 (1.014-1.048)			
L4 VAT density	0.034	0.018	1.035 (1.006-1.064)			
L4 SM density	-0.010	0.541	0.990 (0.958-1.023)			
L4 IMAT density	0.014	0.467	1.014 (0.976-1.054)			
L5 SAI	-0.018	0.012	0.982 (0.968-0.996)			
L5 VAI	-0.022	0.012	0.979 (0.962-0.995)			
L5 SMI	0.013	0.233	1.013 (0.992-1.034)			
L5 IMAI	0.033	0.468	1.034 (0.946-1.130)			
L5 VSR	0.474	0.199	1.607 (0.779-3.314)			
L5 VTR	0.563	0.679	1.756 (0.122-25.329)			
L5 SAT density	0.021	0.021	1.021 (1.003-1.039)	0.026	0.005	1.026 (1.008-1.044)
L5 VAT density	0.021	0.202	1.021 (0.989-1.054)			
L5 SM density	-0.049	0.008	0.952 (0.918-0.987)			
L5 IMAT density	0.010	0.566	1.010 (0.977-1.043)			
Time 2	-	-	-	-	-	-

Variables	Univariate analysis			Multivariate analysis		
	β	*p* value	HR (95% CI)	β	*p* value	HR (95% CI)
L3 SAI	-0.009	0.235	0.991 (0.975-1.006)			
L3 VAI	-0.020	0.005	0.980 (0.966-0.994)			
L3 SMI	0.057	<0.001	1.059 (1.039-1.080)	0.041	<0.001	1.042 (1.023-1.061)
L3 IMAI	-0.044	0.451	0.957 (0.853-1.073)			
L3 VSR	-0.329	0.202	0.719 (0.434-1.193)			
L3 VTR	-1.654	0.106	0.191 (0.026-1.424)			
L3 SAT density	0.020	0.016	1.020 (1.004-1.037)			
L3 VAT density	0.036	0.006	1.036 (1.010-1.063)			
L3 SM density	-0.013	0.383	0.987 (0.957-1.017)			
L3 IMAT density	0.025	0.238	1.025 (0.984-1.069)			
L4 SAI	0.003	0.741	1.003 (0.987-1.019)			
L4 VAI	-0.019	0.042	0.982 (0.964-0.999)			
L4 SMI	-0.014	0.146	0.986 (0.967-1.005)			
L4 IMAI	-0.133	0.076	0.875 (0.755-1.014)			
L4 VSR	0.102	0.767	1.107 (0.564-2.175)			
L4 VTR	-2.550	0.036	0.078 (0.007-0.842)	-3.804	0.004	0.022 (0.002-0.304)
L4 SAT density	0.021	0.023	1.022 (1.003-1.041)			
L4 VAT density	0.054	<0.001	1.055 (1.027-1.084)			
L4 SM density	-0.011	0.492	0.989 (0.957-1.021)			
L4 IMAT density	0.075	<0.001	1.078 (1.034-1.124)			
L5 SAI	-0.008	0.396	0.992 (0.975-1.010)			
L5 VAI	-0.018	0.036	0.983 (0.967-0.999)			
L5 SMI	-0.011	0.311	0.989 (0.969-1.010)			
L5 IMAI	-0.008	0.876	0.992 (0.893-1.101)			
L5 VSR	-0.133	0.718	0.876 (0.426-1.799)			
L5 VTR	-3.015	0.017	0.049 (0.004-0.582)			
L5 SAT density	0.020	0.024	1.020 (1.003-1.038)			
L5 VAT density	0.067	<0.001	1.069 (1.038-1.101)			
L5 SM density	-0.024	0.146	0.976 (0.945-1.008)			
L5 IMAT density	0.033	0.043	1.034 (1.001-1.067)			
Delta	-	-	-	-	-	-
L3 SAI	-0.054	0.448	0.947 (0.824-1.089)			
L3 VAI	-0.108	0.252	0.898 (0.747-1.079)			
L3 SMI	0.116	0.358	1.123 (0.877-1.440)			
L3 IMAI	-0.151	0.137	0.860 (0.705-1.049)			
L3 VSR	-0.129	0.139	0.879 (0.741-1.043)			
L3 VTR	-0.022	0.939	0.978 (0.561-1.706)			
L3 SAT density	0.483	0.337	1.621 (0.605-4.339)			
L3 VAT density	-1.627	0.075	0.197 (0.033-1.176)			
L3 SM density	0.378	0.444	1.460 (0.554-3.847)			
L3 IMAT density	-1.088	0.237	0.337 (0.055-2.049)			
L4 SAI	-0.006	0.772	0.994 (0.954-1.036)			
L4 VAI	0.021	0.663	1.021 (0.930-1.120)			
L4 SMI	-1.691	0.001	0.184 (0.067-0.506)	-2.303	<0.001	0.100 (0.028-0.354)
L4 IMAI	-0.123	0.238	0.884 (0.721-1.085)			

Variables	Univariate analysis			Multivariate analysis		
	β	*p* value	HR (95% CI)	β	*p* value	HR (95% CI)
L4 VSR	-0.143	0.210	0.867 (0.693-1.084)			
L4 VTR	-0.427	0.184	0.653 (0.348-1.225)			
L4 SAT density	0.720	0.193	2.054 (0.694-6.080)			
L4 VAT density	-0.946	0.334	0.388 (0.057-2.649)			
L4 SM density	-0.248	0.644	0.781 (0.273-2.233)			
L4 IMAT density	-1.807	0.050	0.164 (0.027-1.002)			
L5 SAI	0.001	0.980	1.001 (0.912-1.099)			
L5 VAI	0.066	0.505	1.068 (0.880-1.296)			
L5 SMI	-0.651	0.158	0.522 (0.211-1.288)			
L5 IMAI	-0.007	0.667	0.993 (0.962-1.025)			
L5 VSR	-0.353	0.057	0.702 (0.488-1.011)			
L5 VTR	-0.864	0.020	0.422 (0.204-0.873)			
L5 SAT density	0.049	0.935	1.050 (0.319-3.454)			
L5 VAT density	-2.645	0.013	0.071 (0.009-0.574)			
L5 SM density	0.459	0.464	1.582 (0.463-5.409)			
L5 IMAT density	-0.896	0.306	0.408 (0.073-2.271)			
Clinical	-	-	-	-	-	-
Age	0.043	<0.001	1.044 (1.022-1.067)	0.051	<0.001	1.053 (1.026-1.079)
Female			1.000 (Reference)			
Male	0.100	0.739	1.105 (0.613-1.992)			
Disease duration	0.071	0.667	1.074 (0.777-1.483)			
Immunomodulators			1.000 (Reference)			
Biologics	0.497	0.102	1.643 (0.906-2.980)			
Combination	-0.674	0.197	0.510 (0.183-1.420)			
Leukocyte	-0.020	0.732	0.980 (0.872-1.101)			
Hemoglobin	-0.006	0.256	0.994 (0.983-1.005)			
Platelet	0.008	0.003	1.008 (1.003-1.013)	0.007	0.010	1.007 (1.002-1.013)
Hematocrit	-0.019	0.402	0.981 (0.938-1.026)			
C-reactive protein	0.005	0.056	1.005 (1.000-1.011)			
ESR	0.124	<0.001	1.132 (1.080-1.186)	0.105	<0.001	1.110 (1.053-1.171)
Calprotectin	0.003	<0.001	1.003 (1.002-1.004)			
Albumin	-0.050	<0.001	0.951 (0.924-0.979)	-0.044	0.018	0.957 (0.923-0.993)
CTE	-	-	-	-	-	-
Length	0.051	<0.001	1.053 (1.035-1.070)	0.030	0.001	1.030 (1.012-1.049)
Diameter	-0.009	0.812	0.991 (0.922-1.065)			
Proximal	0.008	0.144	1.008 (0.997-1.019)			
Thickness	0.038	0.432	1.039 (0.945-1.142)			

Note: IMAI, intermuscular adipose index; IMAT, intermuscular adipose tissue; SAI, subcutaneous adipose index; SAT, subcutaneous adipose tissue; SMI, skeletal muscle index; SM, skeletal muscle; VAI, visceral adipose index; VAT, visceral adipose tissue; VAR, VAT/SAT ratio; VTR, VAT/total adipose tissue index; HR: Hazards Ratio; CI, confidence interval.

6 Supplementary Material 6: Univariate and multivariate survival analysis for predicting high risk patients (male and female).

Table S8: Univariate and multivariate survival analysis for predicting high risk patients in male cohort.

Variables	Univariate analysis			Multivariate analysis		
	β	*p* value	HR (95% CI)	β	*p* value	HR (95% CI)
Time 1	-	-	-	-	-	-
L3 SAI	-0.006	0.221	0.994 (0.984-1.004)			
L3 VAI	0.001	0.859	1.001 (0.991-1.011)			
L3 SMI	-0.008	0.271	0.992 (0.978-1.006)			
L3 IMAI	0.009	0.904	1.009 (0.872-1.168)			
L3 VSR	0.375	0.162	1.455 (0.861-2.461)			
L3 VTR	-1.849	0.065	0.157 (0.022-1.120)			
L3 SAT density	0.020	0.022	1.020 (1.003-1.038)			
L3 VAT density	-0.007	0.561	0.993 (0.968-1.018)			
L3 SM density	-0.023	0.124	0.978 (0.950-1.006)			
L3 IMAT density	0.013	0.513	1.013 (0.975-1.053)			
L4 SAI	-0.012	0.070	0.988 (0.975-1.001)			
L4 VAI	-0.004	0.488	0.996 (0.984-1.008)			
L4 SMI	0.021	0.005	1.021 (1.006-1.036)			
L4 IMAI	0.059	0.317	1.061 (0.945-1.190)			
L4 VSR	-0.008	0.981	0.992 (0.538-1.832)			
L4 VTR	-1.412	0.211	0.244 (0.027-2.231)			
L4 SAT density	0.022	0.006	1.023 (1.007-1.039)			
L4 VAT density	0.022	0.098	1.022 (0.996-1.048)			
L4 SM density	-0.015	0.339	0.985 (0.956-1.016)			
L4 IMAT density	-0.008	0.665	0.992 (0.956-1.029)			
L5 SAI	-0.014	0.044	0.986 (0.973-0.999)			
L5 VAI	-0.007	0.360	0.993 (0.978-1.008)			
L5 SMI	0.026	0.016	1.027 (1.005-1.049)			
L5 IMAI	0.046	0.276	1.047 (0.964-1.138)			
L5 VSR	0.321	0.377	1.379 (0.676-2.813)			
L5 VTR	-0.435	0.741	0.647 (0.049-8.569)			
L5 SAT density	0.015	0.094	1.015 (0.998-1.032)			
L5 VAT density	0.004	0.802	1.004 (0.972-1.037)			
L5 SM density	-0.042	0.009	0.959 (0.929-0.990)	-0.057	<0.001	0.944 (0.914-0.976)
L5 IMAT density	0.005	0.766	1.005 (0.975-1.035)			
Time 2	-	-	-	-	-	-
L3 SAI	-0.008	0.291	0.992 (0.977-1.007)			
L3 VAI	-0.023	<0.001	0.977 (0.964-0.990)			
L3 SMI	0.055	<0.001	1.057 (1.038-1.076)	0.054	<0.001	1.056 (1.036-1.076)
L3 IMAI	-0.127	0.035	0.881 (0.783-0.991)			
L3 VSR	-0.168	0.482	0.845 (0.529-1.351)			
L3 VTR	-1.293	0.156	0.274 (0.046-1.636)			
L3 SAT density	0.016	0.052	1.016 (1.000-1.032)			
L3 VAT density	0.042	<0.001	1.043 (1.019-1.067)			

Variables	Univariate analysis			Multivariate analysis		
	β	*p* value	HR (95% CI)	β	*p* value	HR (95% CI)
L3 SM density	-0.014	0.342	0.986 (0.959-1.015)			
L3 IMAT density	0.017	0.385	1.017 (0.979-1.058)			
L4 SAI	0.000	0.977	1.000 (0.983-1.017)			
L4 VAI	-0.018	0.044	0.982 (0.964-0.999)			
L4 SMI	-0.007	0.431	0.993 (0.975-1.011)			
L4 IMAI	-0.153	0.040	0.858 (0.741-0.993)	-0.333	<0.001	0.717 (0.600-0.857)
L4 VSR	0.383	0.225	1.466 (0.790-2.721)			
L4 VTR	-2.037	0.060	0.130 (0.016-1.088)			
L4 SAT density	0.027	0.002	1.027 (1.010-1.045)			
L4 VAT density	0.065	<0.001	1.068 (1.041-1.095)			
L4 SM density	-0.021	0.164	0.979 (0.950-1.009)			
L4 IMAT density	0.085	<0.001	1.089 (1.049-1.131)			
L5 SAI	-0.015	0.082	0.985 (0.968-1.002)			
L5 VAI	-0.021	0.005	0.979 (0.964-0.994)			
L5 SMI	-0.007	0.496	0.993 (0.973-1.013)			
L5 IMAI	-0.071	0.177	0.932 (0.841-1.033)			
L5 VSR	0.374	0.222	1.453 (0.798-2.646)			
L5 VTR	-2.549	0.032	0.078 (0.008-0.807)			
L5 SAT density	0.024	0.006	1.024 (1.007-1.042)			
L5 VAT density	0.072	<0.001	1.074 (1.046-1.103)	0.054	<0.001	1.055 (1.029-1.082)
L5 SM density	-0.020	0.197	0.980 (0.951-1.010)			
L5 IMAT density	0.051	<0.001	1.052 (1.022-1.084)			
Delta	-	-	-	-	-	-
L3 SAI	-0.003	0.709	0.997 (0.984-1.011)			
L3 VAI	0.005	0.826	1.005 (0.964-1.047)			
L3 SMI	0.160	0.141	1.174 (0.948-1.453)			
L3 IMAI	-0.017	0.618	0.983 (0.919-1.051)			
L3 VSR	-0.092	0.160	0.912 (0.802-1.037)			
L3 VTR	0.093	0.701	1.097 (0.684-1.759)			
L3 SAT density	0.239	0.680	1.270 (0.408-3.948)			
L3 VAT density	-2.384	0.007	0.092 (0.016-0.516)			
L3 SM density	-0.010	0.982	0.990 (0.415-2.363)			
L3 IMAT density	0.043	0.958	1.044 (0.211-5.174)			
L4 SAI	-0.007	0.741	0.993 (0.950-1.037)			
L4 VAI	0.008	0.876	1.008 (0.909-1.118)			
L4 SMI	-1.278	0.004	0.278 (0.116-0.671)			
L4 IMAI	-0.178	0.119	0.837 (0.669-1.047)			
L4 VSR	-0.096	0.271	0.909 (0.766-1.078)			
L4 VTR	0.158	0.336	1.171 (0.849-1.615)			
L4 SAT density	-0.004	0.994	0.996 (0.343-2.897)			
L4 VAT density	-2.086	0.027	0.124 (0.019-0.793)			
L4 SM density	-0.419	0.421	0.658 (0.237-1.826)			
L4 IMAT density	-2.691	0.002	0.068 (0.012-0.368)			
L5 SAI	-0.011	0.836	0.989 (0.886-1.102)			
L5 VAI	-0.185	0.229	0.831 (0.615-1.123)			
L5 SMI	-0.982	0.036	0.375 (0.150-0.938)			

Variables	Univariate analysis			Multivariate analysis		
	β	*p* value	HR (95% CI)	β	*p* value	HR (95% CI)
L5 IMAI	-0.005	0.720	0.995 (0.969-1.022)			
L5 VSR	-0.026	0.680	0.974 (0.860-1.103)			
L5 VTR	-0.451	0.180	0.637 (0.330-1.232)			
L5 SAT density	-0.267	0.661	0.766 (0.233-2.519)			
L5 VAT density	-3.863	<0.001	0.021 (0.003-0.143)			
L5 SM density	0.324	0.571	1.383 (0.451-4.236)			
L5 IMAT density	-1.803	0.027	0.165 (0.033-0.819)			
Clinical	-	-	-	-	-	-
Age	0.035	<0.001	1.035 (1.015-1.056)	0.038	<0.001	1.038 (1.017-1.060)
Duration	-0.046	0.763	0.955 (0.709-1.287)			
Immunomodulators			1.000 (Reference)			
Biologics	0.390	0.164	1.477 (0.853-2.558)			
Combination	-0.472	0.273	0.624 (0.268-1.451)			
Leukocyte	0.007	0.897	1.007 (0.903-1.123)			
Hemoglobin	-0.002	0.723	0.998 (0.988-1.008)			
Platelet	0.008	<0.001	1.008 (1.003-1.013)			
Hematocrit	-0.010	0.658	0.990 (0.949-1.034)			
C-reactive protein	0.006	0.015	1.006 (1.001-1.011)			
ESR	0.111	<0.001	1.117 (1.069-1.167)	0.070	0.002	1.073 (1.025-1.122)
Calprotectin	0.003	<0.001	1.003 (1.002-1.004)			
Albumin	-0.037	0.009	0.964 (0.938-0.991)			
CTE	-	-	-	-	-	-
Length	0.043	<0.001	1.044 (1.030-1.057)	0.036	<0.001	1.037 (1.022-1.053)
Diameter	-0.023	0.515	0.978 (0.913-1.047)			
Proximal	0.006	0.339	1.006 (0.994-1.018)			
Thickness	-0.005	0.928	0.995 (0.901-1.099)			

Note: IMAI, intermuscular adipose index; IMAT, intermuscular adipose tissue; SAI, subcutaneous adipose index; SAT, subcutaneous adipose tissue; SMI, skeletal muscle index; SM, skeletal muscle; VAI, visceral adipose index; VAT, visceral adipose tissue; VAR, VAT/SAT ratio; VTR, VAT/total adipose tissue index; HR: Hazards Ratio; CI, confidence interval.

Table S9 Univariate and multivariate survival analysis for predicting high risk patients in female cohort.

Variables	Univariate analysis			Multivariate analysis		
	β	*p* value	HR (95% CI)	β	*p* value	HR (95% CI)
Time 1	-	-	-	-	-	-
L3 SAI	-0.014	0.186	0.986 (0.966-1.007)			
L3 VAI	-0.017	0.099	0.983 (0.964-1.003)			
L3 SMI	-0.020	0.228	0.980 (0.948-1.013)			
L3 IMAI	-0.007	0.967	0.993 (0.717-1.375)			
L3 VSR	0.434	0.295	1.544 (0.685-3.483)			
L3 VTR	-0.454	0.801	0.635 (0.019-21.699)			

Variables	Univariate analysis			Multivariate analysis		
	β	*p* value	HR (95% CI)	β	*p* value	HR (95% CI)
L3 SAT density	0.040	0.005	1.041 (1.012-1.070)			
L3 VAT density	0.002	0.920	1.002 (0.958-1.048)			
L3 SM density	-0.016	0.584	0.984 (0.928-1.043)			
L3 IMAT density	-0.018	0.644	0.982 (0.911-1.059)			
L4 SAI	-0.027	0.045	0.973 (0.947-0.999)			
L4 VAI	0.006	0.496	1.006 (0.988-1.024)			
L4 SMI	0.010	0.508	1.010 (0.980-1.041)			
L4 IMAI	-0.171	0.219	0.843 (0.642-1.107)			
L4 VSR	-0.035	0.950	0.965 (0.318-2.930)			
L4 VTR	-2.998	0.123	0.050 (0.001-2.257)			
L4 SAT density	0.044	0.012	1.045 (1.010-1.082)			
L4 VAT density	0.096	<0.001	1.100 (1.044-1.159)			
L4 SM density	0.011	0.719	1.012 (0.950-1.077)			
L4 IMAT density	0.003	0.934	1.003 (0.938-1.072)			
L5 SAI	-0.030	0.031	0.970 (0.944-0.997)			
L5 VAI	-0.046	0.008	0.955 (0.923-0.988)			
L5 SMI	0.018	0.304	1.019 (0.983-1.055)			
L5 IMAI	-0.078	0.380	0.925 (0.777-1.101)			
L5 VSR	-0.131	0.873	0.877 (0.177-4.339)			
L5 VTR	1.799	0.492	6.042 (0.036-1022.336)			
L5 SAT density	0.023	0.116	1.023 (0.994-1.053)			
L5 VAT density	0.068	0.009	1.070 (1.017-1.126)			
L5 SM density	-0.042	0.238	0.959 (0.894-1.028)			
L5 IMAT density	0.006	0.861	1.006 (0.946-1.069)			
Time 2	-	-	-	-	-	-
L3 SAI	-0.023	0.071	0.978 (0.954-1.002)			
L3 VAI	-0.046	<0.001	0.955 (0.931-0.980)	-0.052	0.002	0.949 (0.919-0.980)
L3 SMI	0.059	0.001	1.061 (1.023-1.101)			
L3 IMAI	-0.083	0.496	0.920 (0.725-1.169)			
L3 VSR	-0.347	0.433	0.707 (0.296-1.685)			
L3 VTR	-0.695	0.703	0.499 (0.014-17.915)			
L3 SAT density	0.036	0.015	1.036 (1.007-1.067)			
L3 VAT density	0.056	0.034	1.057 (1.004-1.113)			
L3 SM density	-0.014	0.665	0.986 (0.925-1.051)			
L3 IMAT density	0.057	0.104	1.058 (0.988-1.133)			
L4 SAI	0.014	0.241	1.014 (0.990-1.039)			
L4 VAI	-0.004	0.813	0.996 (0.967-1.027)			
L4 SMI	0.007	0.699	1.007 (0.971-1.046)			
L4 IMAI	-0.215	0.114	0.807 (0.618-1.053)			
L4 VSR	-0.630	0.377	0.533 (0.132-2.157)			
L4 VTR	-2.801	0.170	0.061 (0.001-3.321)			
L4 SAT density	0.046	0.013	1.047 (1.010-1.086)			
L4 VAT density	0.061	0.018	1.063 (1.010-1.117)			
L4 SM density	0.014	0.650	1.015 (0.953-1.080)			
L4 IMAT density	0.038	0.347	1.039 (0.960-1.125)			
L5 SAI	-0.004	0.830	0.996 (0.964-1.030)			

Variables	Univariate analysis			Multivariate analysis		
	β	*p* value	HR (95% CI)	β	*p* value	HR (95% CI)
L5 VAI	-0.011	0.512	0.989 (0.959-1.021)			
L5 SMI	0.018	0.404	1.018 (0.976-1.062)			
L5 IMAI	0.077	0.442	1.080 (0.888-1.312)			
L5 VSR	-0.181	0.825	0.835 (0.168-4.158)			
L5 VTR	-4.315	0.083	0.013 (0.000-1.744)			
L5 SAT density	0.030	0.056	1.031 (0.999-1.063)			
L5 VAT density	0.099	0.002	1.104 (1.037-1.175)			
L5 SM density	-0.041	0.193	0.959 (0.901-1.021)			
L5 IMAT density	0.022	0.490	1.022 (0.961-1.086)			
Delta	-	-	-	-	-	-
L3 SAI	0.025	0.880	1.025 (0.744-1.411)			
L3 VAI	-0.683	0.062	0.505 (0.247-1.033)			
L3 SMI	1.507	0.016	4.513 (1.319-15.442)	3.064	0.005	21.421 (2.471-185.682)
L3 IMAI	-0.135	0.484	0.874 (0.600-1.274)			
L3 VSR	-0.230	0.332	0.795 (0.500-1.264)			
L3 VTR	-0.402	0.498	0.669 (0.209-2.140)			
L3 SAT density	0.570	0.425	1.769 (0.435-7.188)			
L3 VAT density	-1.880	0.242	0.153 (0.007-3.551)			
L3 SM density	-0.053	0.961	0.949 (0.116-7.751)			
L3 IMAT density	-2.658	0.101	0.070 (0.003-1.687)			
L4 SAI	0.163	0.013	1.177 (1.035-1.339)			
L4 VAI	0.048	0.247	1.049 (0.968-1.137)			
L4 SMI	-0.360	0.673	0.698 (0.131-3.713)			
L4 IMAI	-0.028	0.811	0.972 (0.773-1.224)			
L4 VSR	0.040	0.739	1.041 (0.823-1.316)			
L4 VTR	-0.041	0.895	0.960 (0.523-1.761)			
L4 SAT density	0.090	0.943	1.094 (0.092-12.975)			
L4 VAT density	1.778	0.306	5.919 (0.196-178.298)			
L4 SM density	-0.330	0.705	0.719 (0.131-3.961)			
L4 IMAT density	-1.093	0.493	0.335 (0.015-7.608)			
L5 SAI	0.071	0.116	1.074 (0.982-1.174)			
L5 VAI	0.124	0.023	1.132 (1.017-1.259)			
L5 SMI	0.056	0.941	1.057 (0.241-4.639)			
L5 IMAI	-0.017	0.812	0.983 (0.855-1.131)			
L5 VSR	-0.379	0.310	0.684 (0.329-1.423)			
L5 VTR	-1.309	0.097	0.270 (0.058-1.268)			
L5 SAT density	-0.289	0.763	0.749 (0.115-4.894)			
L5 VAT density	0.056	0.974	1.058 (0.036-31.522)			
L5 SM density	-0.028	0.981	0.973 (0.105-9.027)			
L5 IMAT density	-0.728	0.656	0.483 (0.020-11.884)			
Clinical	-	-	-	-	-	-
Duration	0.010	0.969	1.010 (0.605-1.688)			
Immunomodulators			1.000 (Reference)			
Biologics	-0.007	0.990	0.993 (0.354-2.788)			
Combination	-0.822	0.326	0.439 (0.085-2.269)			

Variables	Univariate analysis			Multivariate analysis		
	β	*p* value	HR (95% CI)	β	*p* value	HR (95% CI)
Leukocyte	0.082	0.312	1.086 (0.926-1.273)			
Hemoglobin	-0.010	0.282	0.990 (0.972-1.008)			
Platelet	0.011	0.033	1.012 (1.001-1.022)			
Hematocrit	0.008	0.832	1.008 (0.936-1.086)			
C-reactive protein	0.007	0.119	1.007 (0.998-1.016)			
ESR	0.183	<0.001	1.200 (1.090-1.321)	0.199	0.002	1.221 (1.074-1.388)
Calprotectin	0.003	0.007	1.003 (1.001-1.005)			
Albumin	-0.101	<0.001	0.904 (0.862-0.947)	-0.089	0.002	0.915 (0.866-0.967)
CTE	-	-	-	-	-	-
Length	0.055	0.001	1.057 (1.021-1.093)	0.065	0.002	1.068 (1.025-1.112)
Diameter	0.073	0.361	1.076 (0.919-1.260)			
Diameter Proximal	0.019	0.272	1.019 (0.985-1.055)			
Thickness	-0.032	0.703	0.968 (0.821-1.142)			

Note: IMAI, intermuscular adipose index; IMAT, intermuscular adipose tissue; SAI, subcutaneous adipose index; SAT, subcutaneous adipose tissue; SMI, skeletal muscle index; SM, skeletal muscle; VAI, visceral adipose index; VAT, visceral adipose tissue; VAR, VAT/SAT ratio; VTR, VAT/total adipose tissue index; HR: Hazards Ratio; CI, confidence interval.

7 Supplementary Material 7: Validation of the nomogram.

Figure S4

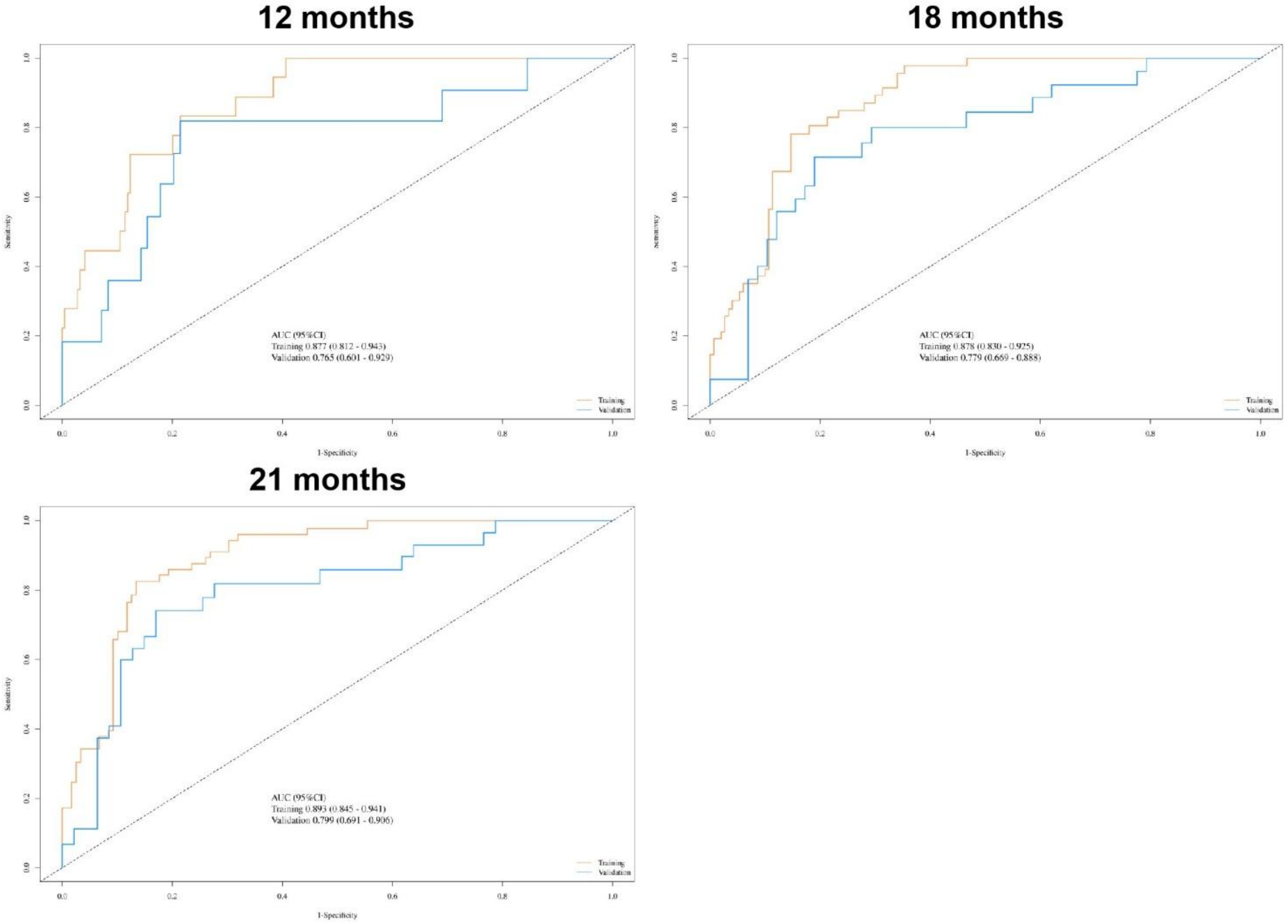

Note: Receiver operating characteristic curves for predicting 12 months, 18 months, and 21 months high-risk groups in the training cohort and validation cohort were shown.

Figure S5

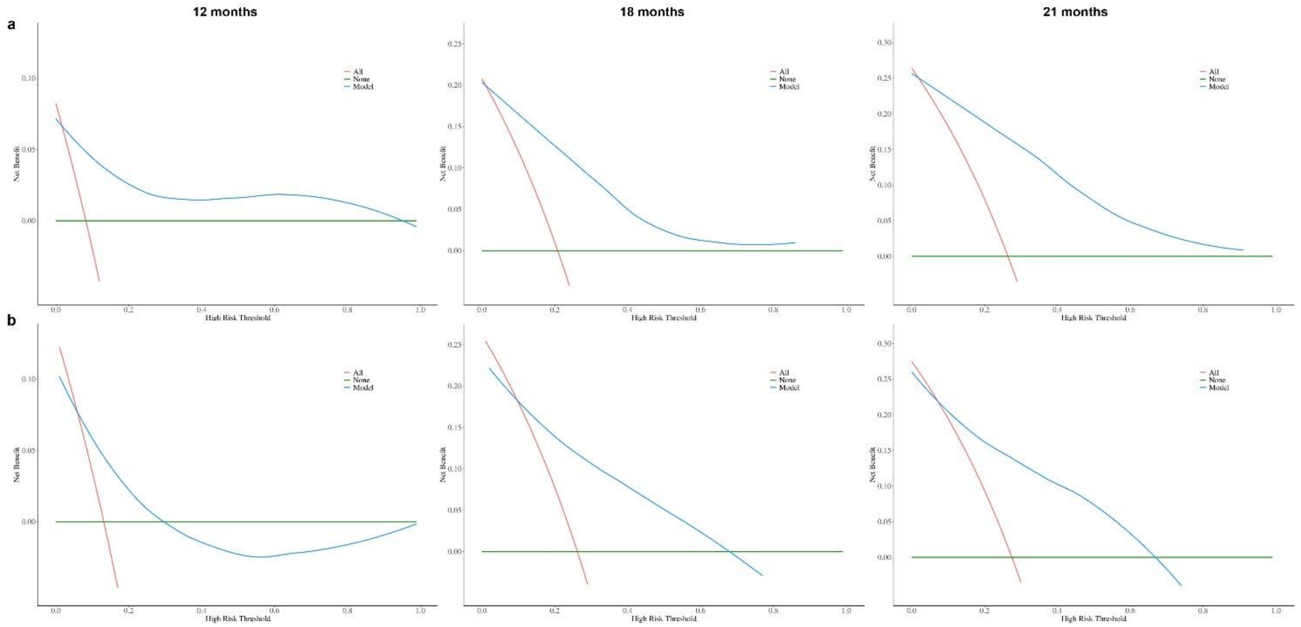

Note: The decision curve of the nomogram in the training cohort (a) and validation cohort (b).

Figure S6

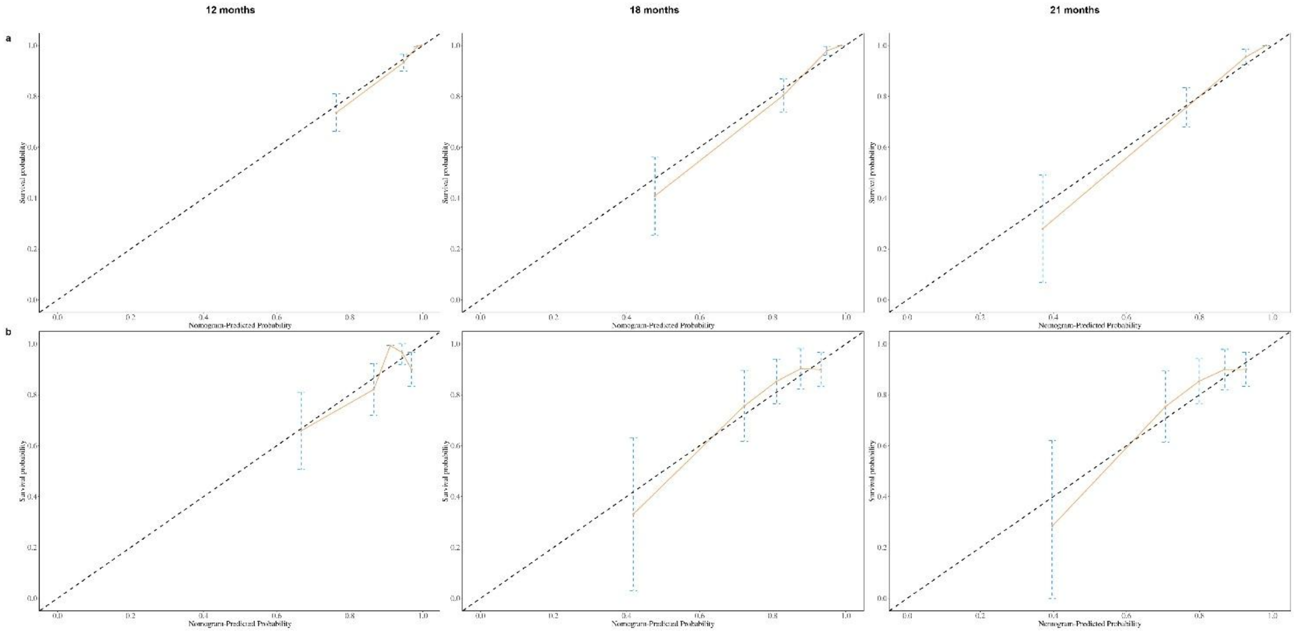

Note: Calibration curves for the nomogram in the (a) training and (b) validation cohorts, derived from 1000 bootstrap resampling iterations.
